# Prevalence and socioeconomic determinants of development delay among children in Ceará, Brazil: A population-based study

**DOI:** 10.1371/journal.pone.0215343

**Published:** 2019-11-05

**Authors:** Luciano Lima Correia, Hermano Alexandre Lima Rocha, Christopher Robert Sudfeld, Sabrina Gabriele Maia Oliveira Rocha, Álvaro Jorge Madeiro Leite, Jocileide Sales Campos, Anamaria Cavalcante e Silva

**Affiliations:** 1 Department of Community Health, Federal University of Ceará, Fortaleza, CE, Brazil; 2 Department of Global Health and Population, Harvard T. H. Chan School of Public Health, Boston, MA; 3 Department of Maternal and Child Health, Federal University of Ceará, Fortaleza, CE, Brazil; 4 ISEC, University Center Unichristus, Fortaleza, CE, Brazil; Monash University, AUSTRALIA

## Abstract

**Objective:**

To assess the prevalence of child development delay and to identify socioeconomic determinants.

**Study design:**

We conducted a population-based cross-sectional study of children 2 to 72 months of age residing in the state of Ceará, Brazil. In total, 3200 households were randomly selected for participation in the study and had child development assessed with the Ages and Stages Questionnaire (ASQ) version 3. Development delay was defined as a score of less than -2 standard deviations below the median of the Brazilian ASQ standard. We present population-level prevalence of delay in five development domains and assess socioeconomic determinants.

**Results:**

A total of 3566 children completed the ASQ development assessment of which 9.2% (95% CI: 8.1–10.5) had at least one domain with development delay. The prevalence of delay increased with age in all domains and males were at higher risk for communication, gross motor and personal-social development delays as compared to females (p-values <0.05). We found robust associations of indicators of socioeconomic status with risk of development delay; increasing monthly income and higher social class were associated with reduced risk of delay across all domains (28,2% in the poorest and 21,2% in richest for any delay, p-values <0.05 for all domains). In addition, children in poor households that participated in conditional cash transfer (CCT) programs appeared to have reduced risk of delay as compared to children from households that were eligible, but did not participate, in CCT programs.

**Conclusions:**

There is a relatively high population-level prevalence of development delay in at least one domain among children 0–6 years of age in Ceará, Brazil. Integrated child development, social support, and poverty reduction interventions may reduce the population-level prevalence of development delay in Ceará and similar settings.

## Introduction

The period from birth to 5 years of age is a critical window for development.[1] Globally, it is estimated that almost 250 million children under five years of age were at risk of failing to reach their full development potential in 2010.[2] Developmental deficits during early childhood may persist and lead to poor schooling achievement and result in reductions in lifetime earnings.[1, 3, 4] As a result, alleviating early life adversity may support attainment health and poverty reduction goal in low- and middle-income countries (LMIC).

There is rapidly growing literature on child development in LMIC settings; however, few studies have evaluated population representative child development and risk factors for delay. Population-level studies in America, Europe and Asia found the prevalence of development delay among children varies from 5 to 15%.[5–8] To our best knowledge, there are no population-based studies in Brazil. Population representative data is needed in Brazil to guide strategies to improve early childhood development to support meeting Sustainable Development Goal target 4.2 which call on countries to ensure all children have access to quality early childhood development, care and pre-primary education.[9]

We present a large cross-sectional and population-representative study of child development among children 0–6 years of age in the state of Ceará, Brazil. We determined the prevalence of children development delay as assessed by the Ages and Stages Questionnaire version 3 and assessed age, sex, and socioeconomic correlates of delay. Our study is intended to inform priority interventions and the need for integration of social protection and poverty-reduction programs to equitably improve child development in Ceará, Brazil.

## Methods

### Study design and population

We analyzed cross-sectional data from the *Pesquisa de Saúde Materno Infantil do Ceará* (PESMIC) study. Full details of the PESMIC study methods can be found elsewhere, as well as all the variable description and full content of the survey.[10] The questionnaire of the research is available as supplementary material S1 and S2 Charts. Briefly, the PESMIC is a population-based study on maternal and child health of preschool children up to 72 months of age living in the state of Ceará, in northeastern Brazil. Population-based surveys were conducted in 1987, 1990, 1994, 2001, 2007 and 2017 using the same methods. For this article, we used data from 2017 which collected child development data.

Ceará is one of the poorest states in Brazil, with a population of 9 million inhabitants living in a semiarid climate. Fortaleza (2.3 million inhabitants) is the capital and urban commercial center of Ceará. The study area also includes rural areas of Ceará where subsistence farming is dominant. *Bolsa Família* is the world’s largest conditional cash transfer (CCT) program, including almost 14 million families or 55 million individuals in Brazil (more than a quarter of the entire population).[11] *Bolsa Família* is a direct income transfer program for families living in poverty (monthly income per capita between R$ 85.01, US$ 22.94, and R$ 170.00, US$ 45.87) and extreme poverty (monthly income per capita of up to R$ 85.00, US$ 22.94), created in 2003, combining several other fragmented social assistance programs. In Ceará, the *Bolsa Família* program's conditionalities include attendance to antenatal care, children under 7 years of age to receive all recommended vaccines and all children attend routine health and growth monitoring visits.[12]. The average grant is about US$75 per month. In Ceará, 1,043,476 families are beneficiaries of the *Bolsa Família* program.

The PESMIC used cluster sampling based on the Brazilian Institute of Geography and Statistics (IBGE) census tracts with stratification between the state capital Fortaleza, and the rural areas. Census tracts were constructed from the division of each municipality into geographic areas with a stable population of 300 families. To ensure the study population was representative, municipalities, census tracts and households were randomly selected, first by randomizing the first participant in an aleatory fashion, followed by stratified selection of municipalities and finally by geographic randomization for selecting the census tract. Once a census tract was defined and its corresponding map obtained, 20 houses were randomly selected, as defined in the first study of 1987. The starting point of the cluster (the first home to be visited) was randomly selected utilizing ArcGIS ® software, GIS Inc. Households were visited consecutively, in a counterclockwise fashion. Shops and abandoned buildings were excluded and replaced; and in the case of absent families, up to three return visits were conducted in an attempt to obtain data.

Data used in the present study was collected by trained interviewers from August to November of 2017, using three questionnaires covering household, maternal and child factors. The questionnaires were reviewed daily by field supervisors to identify and correct errors. During the fieldwork, data of a subsample (10% of children) were reassessed by supervisors for quality control. In the 2017 PESMIC the sample size was defined in 160 census tracts randomly selected. This sample comprised 3200 households. All children from 2 to 72 months old in households were eligible, although only children up to 66 months had their development assessed due to children development questionnaire specifications. In each household, information was obtained about all children through their mother or primary caregiver, and the children’s anthropometric measurements were taken. To account for recall bias, we checked as much information as possible for children booklet, an official document for all Brazilian children, in which the health professionals take notes of the most important characteristics of the children. One was defined as head of household as perceived by the interviewed mother and in case of doubt the one who contributed most with familiar income.

### Child development assessment

To assess children’s development status, we used the Ages and Stages Questionnaire (ASQ) version 3,[13] which was validated in Brazil with different standards for each sex (ASQ-BR) [14] and has been used prior studies.[15] The ASQ consists of a series of questionnaires divided into 20 different age ranges, which varies from two months of amplitude in the first months of life to up to ten months in older children, due to variation in the developmental marks; which seeks to evaluate children aged between 2 and 66 months (five and a half years). Five domains of child development were measured in the ASQ-BR subsections: communication, broad motor coordination, fine motor coordination, problem solving, and personal/social.[13] The questionnaire was applied by interviewers that were trained for 20 hours by medical professionals who were experienced with the ASQ-BR. The interviewers were most graduated health professionals, mainly nurses. The children development was the main outcome variable of this study.

### Socioeconomic factors

We collected data on multiple indicators of socioeconomic status, that were interpreted as predictors of the main outcome. Head of households were asked to report their monthly income in *Reais* (Brazilian currency) and participation status in *Bolsa Família*. Social class was determined by the Brazilian Criteria “Critério Brasil”, which was developed using a representative sample of the family budget research (POF), carried out by the IBGE, classifying 55,970 domiciles based on household assets. This classification classify the inhabitants in seven categories, ranging from E class (poorest) to A1 class (richest) [16] Food insecurity was assessed through the application of the United States Department of Agriculture (USDA) questionnaire modified by the experience of focal groups in Brazil.[17] The instrument consists of 15 central closed questions, with a yes /no response on the experience in the last three months of food insufficiency at its various levels of intensity, ranging from the apprehension that food may be lacking until the experience of passing all day without eating. Each affirmative answer of the questionnaire is equivalent to one point, varying the score from 0 to 15 points, considering the value zero as the safety condition; 1–5 points as mild insecurity; 6–10 points as moderate insecurity and 11–15 points as severe insecurity. We then dichotomized the variable, using as categories no food insecurity and any food insecurity.

Parental education was measured by number of years of formal education. In Brazil, the first four years of school are the basic education, from five to eight years the second basic degree and more than eight the medium and higher education.

### Statistical analysis

First, we tabulated total domain scores for each child using standard ASQ methods. In these methods, each of the six responses for the children evaluation is graded from 0 to10 points, and the sum is compared with the reference population. For the final scores, if more than two items in an area were skipped, that particular domain was not scored. If one or two items in an area were skipped, we provided an adjusted score by calculating the average score for the completed items in that area, then assigning the skipped item(s) the average score.[13] An adjustment was made for children who were born premature and aged up to 24 months, as suggested by ASQ manual. We then standardized development scores, using the formula Z = (X–m) / s, (where X = the value being standardized, m = the mean of the distribution from reference study, s = standard deviation of the distribution from reference study), for children greater than five months of age to the ASQ-BR standard which was created using 45,640 Brazilian children, stratified by age and sex.[18] We used American standardized score cut-offs for children from 2 to 4 months old.[19] As suggested in ASQ manual and in the literature, we considered < - 2 standard deviation as a confirmatory delay and < - 1 standard deviation as risk of delay.[13, 20] Calculated population-prevalence were adjusted for the sampling design that included stratification and cluster-based sampling, and corrections for these two methods were included by informing for each case the strata and the cluster of belonging and adjusting for that variable. We used χ^2^ tests also adjusted for cluster-sampling to assess statistical significance of differences by socioeconomic indicators. Data were entered twice using EpiInfo 2000 and tested for concordance and analyzed using SPSS Version 23 (SPSS Statistics for Windows, Version 23.0. IBM Inc).

### Ethics

Written consent was obtained from participating women. Written consent for child participation was given by their mothers and consent for participating adolescent minors was obtained from parents or guardians. The survey was approved by the Research Ethics Committee in Brazil (Centro Universitário Christus–Unichristus Research Ethics Committee), under number 73516417.4.0000.5049.

## Results

We contacted 3,200 households for participation in the study, of which all agreed to participate, resulting in a sample size of 3,566 children 0–6 years of age. Table 1 presents the demographic and socioeconomic characteristics of the study population. A total of 1,594 (44.6%) children resided in urban areas of Ceará. The mean monthly family income was1090.4 ±1017.9 *Reais* (about US$ 280) and 78% of the sample was in the lowest Brazilian socioeconomic class. In addition, 58% of the children resided in households that were food insecure and 61% of the families participated in conditional cash transfer programs.

**Table 1:** Characteristics of households and participants who completed the Ages and Stages Questionnaire.

Characteristics		N (%) or mean ± standard deviation^1^
Child Sex	Male	1786 (50)
	Female	1779 (49.9)
Residence	Urban	1594 (44.6)
	Rural	1972 (55.3)
Child age in months		31.8 ± 23.1
Monthly income in Reais		1090.4 ±1017.9
Social Class	Lower	2744 (78.4)
	C2	539 (15.4)
	C1	161 (4.6)
	B2	51 (1.4)
	B1	5 (0.1)
	Higher	0 (0)
Food insecurity		1840 (58)
Head of Household education	Up to 4 years	2426 (73.3)
	5–8 years	690 (20.8)
	More than 8 years	191 (5.7)
Participation in CCT programs	Yes, participate	1943 (54.4)
	No, but is eligible	224 (6.2)
	Not eligible	1399 (39.2)

^1^ A total of 3,200 households participated including 3,566 children

The prevalence of development delay (ASQ-3 domain score < -2 SD) by domain, age and sex is presented in Table 2. Overall, the population-level prevalence of communication delay was 2.0% (95 CI: 1.6–2.7), gross motor 3.8% (95 CI: 3.2–4.6), fine motor coordination 2.7% (95% CI: 2.2–3.5), problem-solving 2.8% (95 CI: 2.3–3.5) and personal-social 2.5% (95 CI: 2.0–3.2). We found that the prevalence of delay for all domains was greater for children who were 36–72 months of age as compared to those who were less than 36 months of age (p-values <0.05). In addition, we determined that males were at higher risk for communication, gross motor and personal-social development delays as compared to females (p-values <0.05).

**Table 2:** Population prevalence of development delay by Ages and Stages Questionnaire domain, child age, and child sex in Ceará, Brazil.

			Communication	Gross motor	Fine motor	Problem-solving	Personal-social
		Total N	N% (95% CI)	N% (95% CI)	N% (95% CI)	N% (95% CI)	N% (95% CI)
All children 2 to 66 months of age							
	All	3284	2.0% (1.6–2.7)	3.8% (3.2–4.6)	2.7% (2.2–3.5)	2.8% (2.3–3.5)	2.5% (2.0–3.2)
	Male	1652	2.6% (1.9–3.5)*	4.8% (3.9–6.0)*	3.1% (2.4–4.1)	3.1% (2.4–4.1)	3.2% (2.4–4.2)*
	Female	1632	1.5% (0.9–2.3)*	2.8% (2.0–3.7)*	2.4% (1.7–3.4)	2.5% (1.8–3.4)	1.8% (1.2–2.8)*
0 to 24 months							
	All	1413	1.3% (0.8–2.1)	3.4% (2.6–4.6)	1.1% (0.6–1.8)	1.7% (1.2–2.6)	0.8% (0.5–1.4)
	Male	710	1.2% (0.6–2.3)	3.3% (2.1–5)	0.9% (0.4–1.9)	2.4% (1.5–3.9)*	0.3% (0.1–1.2)*
	Female	713	1.3% (0.6–3)	3.6% (2.5–5.2)	1.3% (0.7–2.4)	1% (0.5–2.1)*	1.3% (0.7–2.5)*
24 to 36 months							
	All	645	2.4% (1.4–3.9)	2.5% (1.4–4.4)	0.5% (0.2–1.5)	1.4% (0.8–2.7)	2.7% (1.7–4.3)
	Male	331	3.7% (2.1–6.5)*	2.2% (1–4.9)	1% (0.3–2.8)	1.6% (0.7–3.6)	4.3% (2.5–7.2)*
	Female	314	1.0% (0.4–2.9)*	2.9% (1.5–5.7)	0.0% (0–0)	1.3% (0.5–3.4)	1.0% (0.4–3)*
36 to 66 months¶							
	All	1226	2.9% (2.2–3.9)	5% (3.9–6.4)	5.9% (4.6–7.6)	4.9% (3.8–6.5)	4.5% (3.5–6)
	Male	611	3.8% (2.6–5.5)	8.2% (6.3–10.7)*	6.9% (5.1–9.3)	5.0% (3.5–7)	6.1% (4.4–8.5)*
	Female	615	2.0% (1.2–3.4)	1.8% (1.1–3.2)*	4.9% (3.4–7.2)	4.9% (3.4–7.1)	3% (1.9–4.7)*

* Statistically significant difference between males and females (p value lower than 0.05).

¶All were statistically significant difference in combined group by age (0–36 and 36–66 months).

We then present the cumulative number of domains per child with developmental delay (< -2 SD domain score) and also for risk of developmental delay (<-1 SD domain score) in Table 3. In total, 9.2% (95%: 8.1–10.5) of all children sampled had at least one domain with development delay. The prevalence of at least one domain with development delay was significantly greater among males (11.0%; 95% CI: 9.5–12.6%) as compared to females (7.5%; 95% CI: 6.1–9.1) (p-value = 0.02). The overall prevalence of risk of developmental delay (<-1 SD) in at least one domain was 24.3% (95% CI: 22.5–26.2) with a significantly higher prevalence among males (28.1%; 95% CI: 25.8–30.5) as compared to females (20.5%; 95% CI: 18.3–22.8) (p-value <0.001).

**Table 3:** Cumulative number of domains with developmental delay per child out of the five Ages and Stages Questionnaire domains (communication, gross motor, fine motor, problem-solving, and personal-social domains) overall and stratified by child sex.

	Total		Boys		Girls		
	N	% (95% CI)	N	% (95% CI)	N	% (95% CI)	P-value for difference by child sex
Number of Domains with Developmental Delay (SD < -2)							
None	2982	90.9 (89.6–92)	1471	89.1 (87.5–90.5)	1511	92.6 (91–94)	0.02
One	211	6.5 (5.6–7.5)	126	7.7 (6.5–9)	85	5.3 (4.2–6.5)	
Two	51	1.6 (1.2–2.1)	28	1.7 (1.2–2.5)	23	1.5 (0.9–2.3)	
Three	22	0.7 (0.5–1.1)	15	1 (0.6–1.6)	7	0.5 (0.3–0.9)	
Four	5	0.2 (0.1–0.4)	3	0.2 (0.1–0.6)	2	0.2 (0.1–0.5)	
Five	12	0.4 (0.3–0.7)	8	0.5 (0.3–1)	4	0.3 (0.1–0.7)	
Number of Domains with Risk of delay (SD < -1)							
None	2486	75.8 (73.9–77.6)	1187	72 (69.6–74.3)	1299	79.6 (77.3–81.8)	<0.001
One	481	14.7 (13.4–16.1)	278	16.9 (15.1–18.8)	203	12.5 (10.9–14.3)	
Two	184	5.7 (4.9–6.6)	108	6.6 (5.4–8)	76	4.7 (3.8–5.9)	
Three	73	2.3 (1.8–2.9)	42	2.6 (1.9–3.5)	31	1.9 (1.4–2.8)	
Four	38	1.2 (0.9–1.6)	21	1.3 (0.9–2)	17	1.1 (0.7–1.7)	
Five	20	0.7 (0.4–1)	14	0.9 (0.5–1.5)	6	0.4 (0.2–0.9)	

We present the prevalence of development delay by socioeconomic characteristics in Table 4. We found that increasing monthly income quintile was associated with reduced risk of delay in the communication, gross motor and fine motor domains and for delay in any domain (p-values <0.05). In addition, social class was associated with gross motor and fine motor delays (p-values <0.05), while food insecurity was associated with communication delay (p-value = 0.02). Among individuals eligible for conditional cash transfers, it appeared those who participated had lower risk of development delay in communication, gross motor and personal-social domains; however, we tested specifically the difference between those who participated and those who did not participate and the results did not reach statistical significance (p-values > 0.05). There was no association of the head of household education with development delay in any domain. Similar results were found for risk of development delay, presented in Table 5.

**Table 4:** Prevalence of child development delay by domain stratified by socioeconomic factors.

		Communication		Gross motor		Fine motor		Problem solving		Personal social		Any delay	
	N total	N% (95 CI%)	p value	N% (95% CI)	p value	N% (95% CI)	p value	N% (95% CI)	p value	N% (95% CI)	p value	N% (95% CI)	p value
Residence			0.26		0.98		0.28		0.10		0.44		0.22
Urban	1594	2.4% (1.5–3.7)		3.8% (2.9–5.0)		3.1% (2.2–4.4)		3.4% (2.6–4.4)		2.8% (2.0–3.9)		10.0% (8.2–12.2)	
Rural	1972	1.8% (1.3–2.4)		3.8% (2.9–4.9)		2.4% (1.8–3.3)		2.4% (1.7–3.3)		2.3% (2.0–3.2)		8.5% (7.1–10.1)	
Household monthly income in quintiles†			0.02		0.05		0.02		0.17		0.26		0.02
1st—Poorest	638	3.0% (2.0–4.66)		4.9% (3.5–6.8)		4.3% (2.8–6.4)		3.7% (2.3–5.7)		3% (1.8–5.1)		11.3% (8.8–14.4)	
2nd	654	2.6% (1.6–4.3)		3.3% (2.2–4.9)		3.3% (2.2–4.9)		3.9% (2.7–5.6)		3.3% (2.1–5)		10.2% (7.9–13.2)	
3rd	624	2.3% (1.4–3.7)		5.2% (3.8–7.1)		2.3% (1.4–3.9)		2.1% (1.3–3.5)		3.3% (2–5.4)		9.3% (7.2–12.0)	
4th	672	1.1% (0.5–2.2)		3.3% (2.2–5)		1.7% (1–2.9)		2.3% (1.4–3.7)		1.8% (1–3.2)		7.9% (6.1–10.1)	
5th- Richest	578	1.1% (0.5–2.3)		2.5% (1.5–4)		2% (1.1–3.4)		2.3% (1.3–4)		1.8% (1–3.2)		6.4% (4.8–8.5)	
Social class†			0.39		0.008		0.03		0.07		0.32		<0.001
Lower	2525	2.3% (1.8–3.1)		4.6% (3.8–5.6)		3.3% (2.6–4.2)		3.3% (2.7–4.2)		2.9% (2.2–3.7)		10.5% (9.1–12.0)	
C2	495	1.7% (0.9–3.2)		1.5% (0.7–2.9)		0.9% (0.4–2.2)		1.1% (0.4–2.9)		1.9% (0.9–3.7)		4.6% (3.0–7.0)	
C1	149	0.7% (0.1–4.7)		2.1% (0.7–6)		2.1% (0.7–6.3)		2.7% (1.1–7)		0.7% (0.1–4.7)		6.0% (3.2–11.1)	
B2 / B1	51	0% (0–0)		0% (0–0)		0% (0–0)		0% (0–0)		4.1% (1–15.6)		4.1% (1.0–15.6)	
Food insecurity			0.02		0.18		0.73		0.34		0.93		0.02
No	1212	1.2% (0.7–2)		3.2% (2.3–4.3)		2.4% (1.7–3.6)		2.4% (1.6–3.5)		2.4% (1.6–3.7)		7.4% (6.0–9.1)	
Yes	1705	2.4% (1.8–3.3)		4.0% (3.1–5.2)		2.6% (1.9–3.7)		2.9% (2.2–3.9)		2.4% (1.8–3.2)		9.7% (8.3–11.4)	
Head of household education†			0.32		0.81		0.36		0.85		0.52		0.26
Up to 4 years	2240	1.9% (1.3–2.8)		3.7% (2.9–4.6)		2.6% (1.9–3.5)		2.6% (2–3.5)		2.5% (1.9–3.3)		8.4% (7.2–9.8)	
5–8 years	629	2.8% (1.7–4.4)		4.2% (2.7–6.3)		2.9% (1.8–4.6)		3.1% (2–4.7)		3.1% (2–4.8)		10.5% (8.1–13.4)	
More than 8 years	181	1.2% (0.3–4.5)		3.9% (1.9–8)		4.5% (2.3–8.5)		2.8% (1.2–6.5)		1.7% (0.6–5.1)		9.9% (6.3–15.3)	
Eligibility and participation in CCT^1^ programs			0.03		0.10		0.02		0.20		0.007		< 0,001
Eligible and participates	1811	2.4% (1.8–3.3)		4.2% (3.3–5.2)		3.3% (2.5–4.3)		3.3% (2.5–4.3)		3% (2.2–4.1)		10.5% (8.9–12.3)	
Eligible, but does not participate	204	3.5% (1.8–6.8)		6.4% (3.2–12.4)		4% (2.1–7.5)		3.5% (1.8–6.8)		5% (2.8–8.7)		13.2% (8.8–19.4)	
Not eligible	1269	1.4% (0.9–2.2)		3% (2.3–4.1)		1.9% (1.2–2.8)		2.3% (1.6–3.2)		1.6% (1.1–2.5)		6.6% (5.4–8.1)	

† p-value for trend

^1^CCT–Conditional cash transfer

**Table 5:** Prevalence of risk of child development delay by domain stratified by socioeconomic factors.

		Communication		Gross motor		Fine motor		Problem solving		Personal social		Any delay	
	N total	N% (95 CI%)	p value	N% (95% CI)	p value	N% (95% CI)	p value	N% (95% CI)	p value	N% (95% CI)	p value	N% (95% CI)	p value
Residence			0.06		0.54		0.10		0.16		0.36		0.07
Urban	1594	7.4% (6–9.1)		8.2% (6.6–10.1)		9.1% (7.5–11)		9.5% (7.9–11.3)		10% (8.6–11.5)		26.2% (23.3–29.3)	
Rural	1972	5.7% (4.7–6.9)		7.4% (6–9.2)		7.3% (6.1–8.7)		8% (6.8–9.4)		9.1% (7.8–10.5)		22.8% (20.6–25.1)	
Household monthly income in quintiles†			0.01		0.06		0.04		0.004		0.49		0.05
1st—Poorest	638	8.4% (6.3–11)		9.3% (7.1–12)		10.1% (7.9–12.8)		10.9% (8.5–13.9)		10.1% (7.8–13)		28.2% (24.5–32.1)	
2nd	654	7.1% (5.2–9.5)		7.7% (5.7–10.2)		9.1% (6.9–11.8)		11.2% (8.8–14.2)		9.8% (7.7–12.5)		25.7% (22–29.8)	
3rd	624	6.8% (5–9.1)		9.5% (7.2–12.4)		8.2% (6.3–10.7)		7.4% (5.5–10)		10.9% (8.7–13.7)		24.1% (20.8–27.8)	
4th	672	3.6% (2.4–5.4)		6.2% (4.5–8.3)		6.4% (4.8–8.6)		6.7% (5–9)		8.4% (6.4–10.9)		22.2% (19–25.8)	
5th- Richest	578	6.1% (4.4–8.5)		6.1% (4.3–8.5)		6.1% (4.4–8.3)		6.5% (4.9–8.5)		8.4% (6.2–11.1)		21.2% (17.9–24.9)	
Social class†			0.002		0.22		0.01		<0.001		0.18		<0.001
Lower	2525	7.3% (6.3–8.4)		8.2% (7.1–9.6)		9.1% (7.9–10.4)		9.8% (8.6–11.2)		10.2% (9.1–11.5)		26.1% (24.1–28.3)	
C2	495	3.9% (2.5–6.1)		6.3% (4.5–8.8)		4.7% (3.1–7.1)		4.5% (3–6.8)		7.1% (5–10.1)		17.8% (14.4–21.9)	
C1	149	2.7% (1–7.1)		8.1% (4.7–13.7)		4.8% (2.3–9.6)		4.7% (2.3–9.6)		6.8% (3.8–11.9)		19.6% (14–26.9)	
B2	51	4.1% (1.1–14.8)		2.1% (0.3–13.8)		4.1% (1–15.4)		2.1% (0.3–13.3)		10.3% (4.5–21.7)		20.5% (10.8–35.3)	
Food insecurity			0.18		0.05		0.04		0.01		0.09		0.02
No	1212	5.4% (4.2–7)		6.2% (4.8–7.8)		6.6% (5.3–8.1)		6.8% (5.5–8.4)		8.1% (6.7–9.6)		21.4% (19–23.9)	
Yes	1705	6.6% (5.6–7.8)		8.1% (6.7–9.8)		8.6% (7.3–10.2)		9.5% (8.2–11)		9.7% (8.5–11.1)		24.9% (22.8–27.1)	
Head of household education†			0.01		0.89		0.70		0.38		0.16		0.05
Up to 4 years	2240	5.6% (4.6–6.7)		7.5% (6.4–8.9)		7.5% (6.4–8.9)		7.9% (6.6–9.3)		9.2% (8.1–10.4)		22.9% (20.9–25)	
5–8 years	629	9% (7.1–11.3)		8% (5.8–10.9)		8.2% (6.3–10.6)		9.6% (7.4–12.3)		11% (8.8–13.8)		27.3% (24–30.8)	
More than 8 years	181	6.1% (3.4–10.8)		8.3% (5–13.7)		8.9% (5.7–13.5)		9.4% (5.8–15)		6.7% (3.8–11.6)		26% (20.1–33)	
Eligibility and participation in CCT^1^ programs			0.03		0.20		< 0.001		0.001		0.15		0.002
Eligible and participates	1811	7.1% (5.9–8.5)		7.9% (6.6–9.5)		9.5% (8.2–11)		10.4% (9–12.1)		10% (8.6–11.7)		26.5% (24.1–28.9)	
Eligible. but does not participate	204	9.4% (5.8–14.9)		10.8% (6.8–16.9)		10.9% (7–16.5)		8.4% (5–13.8)		11.8% (8.2–16.7)		27.1% (21.2–34)	
Not eligible	1269	5% (3.9–6.5)		7% (5.6–8.7)		5.7% (4.5–7.2)		6.1% (4.9–7.7)		8.3% (7–9.9)		20.8% (18.4–23.4)	

† p-value for trend

^1^CCT–Conditional cash transfer

## Discussion

We conducted a population-based study of development delay among children 2 months to 6 years of age in Ceará, Brazil. We found that 2–3% prevalence of development delay within each of the five domains and a 9.2% prevalence of development delay in at least one of the five domains assessed. Within each domain, the percent of children with delay was comparable to the Brazilian reference population as the percent of children < -2 SD is expected to be 2.5%. Nevertheless, we found that within Ceará, older children were at increased risk of delay across domains and male children had greater risk of delay in the gross motor, problem-solving, and personal-social domains. We also found that lower socioeconomic status, as assessed by monthly income and social class, was strongly associated with increased risk of development delay. In addition, there was some indication of lower prevalence of delay in poor families that participated in CCT programs as compared to those who were eligible but did not participate.

The prevalence of development delay in any of the five development domains in our study (9.2%) which is in line with existing literature in other settings.[5, 6, 8] In non-population representative samples in Brazil, the prevalence of development delay was reported to range from 21.4% to 46.3%, using different appraisal tools and in different ages.[21, 22] To the best of our knowledge this is the first comprehensive population-based study of the risk of child development delay in Brazil. Brazil has a development monitoring program that is estimated to achieve only 4.6% detection rates of cases and referral in some states.[21] Thus, the relatively high population prevalence of any delay and risk of delay and the low reach of monitoring programs suggests the need for evaluating the implementation of current child development policies in Ceará.

We also identified that male children in Ceará had a higher prevalence of delay in the motor, problem-solving, and personal-social domains as compared to females. Although the standards for males and females in ASQ are different, and this may represent a finding only from Ceará deviation from normal curve distribution, this is in line with other studies that identified higher risk of suboptimal development, particularly in verbal and reading abilities, for males as compared to females.[5, 23–28] We are not able to determine mechanisms leading to the sex-differences but we hypothesize differences in parental stimulation by sex and age in Brazilian culture may be a contributor[29]. In our study, the magnitude of the difference between males and females appeared to increase after 36 months of age. By 3 years of age, children follow parental instructions and may partake in different play activities (understood as recreational and interactional activities between parents and children and with other children of the same age) that can lead to sex differences[27]. In some studies, greater access to healthcare and child development services has been proposed to explain the higher prevalence of child development delay among males; however, assessment with the age and sex standardized ASQ-BR in this study does not support this mechanism. As a result, child development interventions that have some degree of tailoring by child sex may provide greater impact in this setting.

In this study we also found that social class, monthly household income and food security were robustly associated with development delays. There is a large literature on the link between poverty and child development.[30–33] Poverty may impact the child development in through a multitude of direct and indirect pathways. Poverty can directly lead to family stress and parental deprivation that leads to suboptimal development outcomes.[34] The chronification of poverty is more strongly associated with poor development than the acute poverty exposure, due to the sustained effect of this stress[35]. In support, in our study we found that social class, had a stronger association with development and with risk of delay as compared to monthly income in some domains.[36, 37] This is particularly important because the home environment is more closely linked to the cognitive development of young preschool children than to that of school‐age children.[31] In addition, poorest children often have less access to schools and to stimulating environments.[38] In Brazil two cohorts identified an association with family income and child development.[22] As a result, there is convincing evidence that targeting low SES households will be essential to improve population-level child development and reaching SDG target 4.2.

Consistent with the SES findings, we found that children who resided in households that were not eligible for CT programs had lower risk of delay than those who lived in eligible households. Nevertheless, we also found that among CT eligible households, children whose family participated in the programs tended to have lower risk of delay as compared to those whose family did not participate. This suggests that there may be some effect of the CT programs on child development outcomes; however, our observational analyses cannot be considered causal. The evidence on the effect of conditional and unconditional cash transfers on child development and general health outcomes is mixed[38, 39]. In a study of *Oportunidades* program in Mexico, participating in the conditional cash transfer programs was associated with better cognition and language development and this finding was confirmed in Nicaragua and Uganda. Conditional cash transfers like *Bolsa Familia* may create positive externalities for improved child development among low SES households; however, additional research is needed to document if the existing CT programs provide child development benefits in Brazil.

This study has some limitations. First, the observational and cross-sectional design of the study does not afford analysis of child development trajectories over time and does not allow for direct determination of causal relationships. Also, statistical adjustments for sample designing must be performed and multivariate analysis were not performed. In addition, we used the ASQ-3, which is a validated screening tool, but is not diagnostic for child development delay, and have specific standard for males and females, which implies in little generalization for comparisons using gender variable. Further, while the study was designed to be population representative of children in the State of Ceará, the prevalence of delay and determinants are not likely generalizable to all Brazilian children.

## Conclusions

Overall, we found that ~10% of children under 6 years of age in Ceará, Brazil had delay in at least one development domain. In addition, the prevalence of delay increased with age which supports the need for early intervention. Males were also at higher risk for delay than females which may have some programmatic and relevance to design of stimulation and play interventions. We also found some evidence that cash transfer programs may ameliorate some of the risk of development delay among children in poor households. As a result, integrated child development interventions that also address underlying poverty-related risk factors and inequity may significantly reduce the population prevalence of development delay in Ceará and similar settings. Implementation research is needed to determine how to best deliver comprehensive child development interventions that are integrated with social protection and poverty reduction interventions.

## Supporting information

S1 Chart Questionnaire used in the research, translated to English. (DOCX)

S2 Chart Questionnaire used in the research, original. (DOC)

## ABBREVIATIONS

PESMIC: Pesquisa de Saúde Materno Infantil do Ceará
IBGE: Brazilian Institute of Geography and Statistics
USDA: United States Department of Agriculture
ANC: Antenatal care
LMIC: low- and middle-income countries
SES: socioeconomic status
SDG: Sustainable development goals
CTP: Cash Transfer Program
