# Supplementary material for: Prevalence and socioeconomic determinants of development delay among children in Ceará, Brazil: A population-based study
Source: PLoS One. 2019 Nov 5;14(11):e0215343. doi: 10.1371/journal.pone.0215343 (PMC6830766; doi:10.1371/journal.pone.0215343)
Supplement: S1 Chart — (DOCX) [file pone.0215343.s001.docx]

| UFC/ Unichristus/ SMS/sesa/ funcap-CNPq  **VI Maternal-infant Health survey in CEARÁ-2017** | HOUSEHOLD INFORMATION |
| --- | --- |

| 01. Municipality [MUN]: _____________ ________________________________________________________________________________ | | | | | 02. District/District [DISBA]: ____________________________________ | |
| --- | --- | --- | --- | --- | --- | --- |
| 03. Municipality Code: [COMUN]: _____ | | | | | 04. IBGE sector [SETOR]: ______*(****4 last Nos. MAP)*** | |
| 05. Sector Zone [ZONE]: 1-Rural2 – Urbana | | | | | 06. House No. [CASNO]: ______ ***(No. of*** the ***sector***sequence***)*** | |
| Address: | Street/Av. ________________________________________________________________________________________________............ ___________ District: ___________________________ .......... | | | | | |
|  | | | | | | |
| 07. Respondent of the questionnaire: respon | | | | | | Mother/wife 1  Father/Spouse 2  Grandpa/Grandmother 3  Child (a) 4  Other: _____________ _ __________________________ 5 in the |
| 08. All in all, how many people live in the house?[MORA] | | | | | | People: _____ |
| 09. How many women from 10 to 49 years old live in the house?[Muan] | | | | | | Women: ___ |
| Ten, ten. How many children under the age of 6 live in the house? [Crian]  *(Children up to 5 years, 11 months and 29 days)* | | | | | | Children: ___ |
| I'm 11. Who's the head of the family? [CHEF]  ***(If in doubt, mark the one that most contributes to the REnda Familiar)*** | | | | | | The pai 1  The mother 2  O Grandpa/grandma 3  Child (a) 4  Other: _______________ ____________________ 5........ |
| I'm 12. Until what year (series) has the head of the family studied at school?*(Passed from year)*  BE  ***(If you have a higher level, note how many years, in ' series ', and 3rd. Degree)*** [degree] | | | | | | ___ Series  ___ Degree |
| Onethree. Does the family possess how many of these goods at home? BA13  ED13  ***(If not, annotate 0 (zero).*** AU13  MC13  LL13  GE13  FZ13  LR13  DV13  MO13  MT13  SR13 | | | | | | Bathrooms ___  Household Employees ___  Automobiles ___  Microcomputer ___  Dishwashers ___  Refrigerator ___  Freezer ___  Wash clothes ___  DVD ___  Micro-Waves ___  Motorcycle ___  Clothes Dryer ___ |
| Fourteen of them. Which of these resources (computer science) does the family have at home?  Internet [IN14]  Notebook [NB14]  Tablet [TA14]  Cellphone (phone only) [CE14]  Smartphone ( touch screen PHONE) [SM14] | | | | | | 1 – Yes 2 – No  1 – Yes 2 – No  1 – Yes 2 – No  1 – Yes 2 – No  1 – Yes 2 – No |
| Onefive. Where does the water used to drink come from? To DRINK | | | | | | Encanada, Indoors 1  Encanada, away from home 2  Cistern 3  Fountain 4  Cacimba 5  River/açude/Lagoa 6  Water mineral 7  Other: _______________ __________________________ 8...... |
| Onesix. What kind of house toilet? [PRIV] | | | | | | Sanitary com discharge 1  Sanitary sin discharge 2  Little House com fossa 3  Fenced hole 4  You don't have 5 |
| 17. What is the type of pavement on the street where the family lives? Pav | | | | | | Asphalt 1  Pavment 2  Earth 3  Other: ______________________________ (4) |
| Oneeight. Does the family participate in the Bolsa familia program? [Bag] | | | | | | Yes, it gets the scholarship 1  Yes, you registered, but you don't get the scholarship 2  ***(If not, skip to Q20)🡪***Not 3 |
| Onenine. If yes, how much did you receive from the Bolsa Familia in the last month? Bolsq | | | | | | R $ ______, 00 |
| Twenty, 20.**In all**, Q Did the family people win last month? [REND]  *(Including family allowance, pension, retirement, etc.)* | | | | | | R $ _____. ______, 00 |
| Twoone. Does anyone in the family have health Insurance (insurance)? [CONV]  (Unimed, IPM, etc.) | | | | | | Yes, paid by the company 1  Yes, I pay for the family 2  Not 3 |
| 22. If yes, what is the name of the plan?CONVN  OUT22 OU22Q | | | | | | Unimed 1  Hap Life 2  Free Life 3  GEAP 4  IPM 5  ISSEC 6  BRadesco Sspecialised 7  AMIL 8  Other: _______________ __________________________ 9...... |
| 23. If yes, how many family members are covered by the plan?CONVQ | | | | | | All people 1  One part: No. of people: _____ |
| 24. last time someone in the family got sick, who did you consult with?  CONSQ  OU24Q | | | | | | Hospital Physician 1  Physician at POST (center) of Health 2  Physician DTO UPA 3  Cuban Physician 4  Health Plan Physician 5  Private physician (paid from own pocket) 6  Other professional: ___________________________________7....... |
| 25. has anyone in the family (who lives in the house) ever tried to do exams without feeling anything/without being sick in the last 12 months (C*heck-up*)?CHECK  CHECKQ method | | | | | | Yes 1  Who? __________________________________  *(Kinship with the child)* 2 – No |
| Twosix. V. Do you know the family health program? Have you ever been visited by someone on the show? [PSF] | | | | | | Yes, family has already been visited at home 1  Yes, PSF in the area, but family not visited 2  Yes, you heard about 3  You don't know 4 |
| Twoseven. Has anyone in the family ever had any of these diseases?  AMI myocardial infarction (heart)  Stroke thecerebral vascular (stroke, thrombosis)  CAN Cancer (type: _____________ _ ____________)  ORT27 OU27Q Another serious illness: ________________________.......... | | | | | | 1 – Yes 2 – No 3 – Do not know  1 – Yes 2 – No 3 – Do not know  1 – Yes 2 – No 3 – Do not know  1 – Yes 2 – No 3 – no Satand |
| **Questions about Insecurity TOlimentar (Ebia) – All issues relate to the last 3 months** | | | | | | |
| 28.01. In the last 3 months,have the speakers been worried that food would end before they can buy or receive more food?Com How often did this happen?  IA01 | | | | Yes, quase every day 1  Yes, theome of Days2  Yes, but only 1 or 2 days 3  Not 4  NS/nQR 5 | | |
| 28.02. In the last three months, were theLiments finished before the villagers had the money to buy more food?  How often did this happen?  IA02 | | | | Yes, quase every day 1  Yes, theome of Days2  Yes, but only 1 or 2 days 3  Not 4  NS/nQR 5 | | |
| 28.03. In the last 3 months,have thespeakers been out of money to have a healthy and varied diet?  How often did this happen?  IA03 | | | | Yes, quase every day 1  Yes, theome of Days2  Yes, but only 1 or 2 days 3  Not 4  NS/nQR 5 | | |
| 28.04. In the last 3 months, Mspeakers have eaten only a few foods they still had, because the money ended?  How often did this happen?  IA04 | | | | Yes, quase every day 1  Yes, theome of Days2  Yes, but only 1 or 2 days 3  Not 4  NS/nQR 5 | | |
| 28.05. In the last 3 months, has theany resident 18 years or older ever decreased the amount of food in meals or ceased to make a meal because there was no money to buy food?  How often did this happen?  IA05 | | | | Yes, quase every day 1  Yes, theome of Days2  Yes, but only 1 or 2 days 3  Not 4  NS/nQR 5 | | |
| 28.06. In the last three months, theany resident of 18 years or older has ever eaten less because there was no money to buy food?  How often did this happen?  IA06 | | | | Yes, quase every day 1  Yes, theome of Days2  Yes, but only 1 or 2 days 3  Not 4  NS/nQR 5 | | |
| 28.07. In the last 3 months, theany resident of 18 years or older has ever felt hungry but did not eat because there was no money to buy food?  How often did this happen?  IA07 | | | | Yes, quase every day 1  Yes, theome of Days2  Yes, but only 1 or 2 days 3  Not 4  NS/nQR 5 | | |
| 28.08. In the last 3 months, theany resident of 18 years or older has lost weight because it has not eaten enough food due to lack of money to buy food?  Qbeen such weight did he lose?  IA08 | | | | Yes little weight 1  Yes, some weight 2  Yes, Much weight 3  Not 2  NS/nQR 5 | | |
| 28.09. In the last 3 months, is theany resident 18 years old or older ever made just one meal or stayed a whole day without eating because there was no money to buy food?  How often did this happen?  IA09 | | | | Yes, quase every day 1  Yes, theome of Days2  Yes, but only 1 or 2 days 3  Not 4  NS/nQR 5 | | |
| 28.10. In the last 3 months, theany resident under **18 years** of age has ever ceased to have a healthy and varied diet because there was no money to buy food?  How often did this happen?  IA10 | | | | Yes, quase every day 1  Yes, theome of Days2  Yes, but only 1 or 2 days 3  Not 4  NS/nQR 5 | | |
| 28.11. In the last three months, theany dweller under **18 years** of age has not eaten enough food because there was no money to buy food?  How often did this happen?  IA11 | | | | Yes, quase every day 1  Yes, theome of Days2  Yes, but only 1 or 2 days 3  Not 4  NS/nQR 5 | | |
| 28.12. In the last 3 months,any dweller under **18 years** of age decreased the amount of food in meals because there was no money to buy food?  IA12 | | | | Yes, quase every day 1  Yes, theome of Days2  Yes, but only 1 or 2 days 3  Not 4  NS/nQR 5 | | |
| 28.13. In the last three months, theany resident under **18 years** of age has ever stopped making a meal because there was no money to buy food?  How often did this happen?  IA13 | | | | Yes, quase every day 1  Yes, theome of Days2  Yes, but only 1 or 2 days 3  Not 4  NS/nQR 5 | | |
| 28.14. In the last three months, theany dweller under **18 years** of age has ever felt hungry but didn't eat because there was no money to buy food?  How often did this happen?  IA14 | | | | Yes, quase every day 1  Yes, theome of Days2  Yes, but only 1 or 2 days 3  Not 4  NS/nQR 5 | | |
| 28.15. In the last three months, theany dweller under **18 years** of age has ever spent a whole day without eating because there was no money to buy food?  How often did this happen?  IA15 | | | | Yes, quase every day 1  Yes, theome of Days2  Yes, but only 1 or 2 days 3  Not 4  NS/nQR 5 | | |
| The 29th. Has a person died in the family house in the last 12 months? MOR | | | Yes 1  Not 2 | | | |
| Three0. If YES, who was (RAM) this person (s)?  *(Kinship in relation to the child)* | | P 30.1 Parentesco: _______________ I 30.1 Age: _______ C 30.1 cause: ________________________......  P 30.2 Parentesco: ______________ I 30.2 Age: ____ C 30.2 cause:____________________________.......  P 30.3 parentesco: _______________ I 30.3 Age: _______ C 30.3 cause:________________________...... | | | | |
| Threeone.The family moved from home in the last12 of the months?  Mud | | | | | | Yes 1  Not 2 |
| 32. If yes, Quantas times moved? | | | | | | Times ___ |
| Threethree. In the last 12 months,has therebeen any of these problems in your family?  MO33 death of family person or family close  AC33 accident or serious illness in the family  SE33 separation or divorce  DI33 child Custody dispute/alimony  PE33 someone lost their job  PR33 someone was arrested  AP33 Lack of family/ Friends Support  OUT33 OU33Q Other:_______________________________ __________________ ......... | | | | | | 1 – Yes 2 – No  1 – Yes 2 – No  1 – Yes 2 – No  1 – Yes 2 – No  1 – Yes 2 – No  1 – Yes 2 – No  1 – Yes 2 – No  1 – Yes 2 – No |
| 34.In your family,there has been or has beenfights, discussing another type of violenceat Home? (Domestic violence)Viol | | | | | | Yes 1  Not 2 |
| 35.In The last 12 months, which of theseKindS of problemshas there been at home?  DI35 discussion, mouthwatering, cusing  AG35 Physical Aggression  EM35 arrive intoxicated, drugged  EX35 Expel someone from home  OUT35 OU35Q Other: ___________________________ _ _ _ _ _ _ _ _ _ _ _________ | | | | | | 1 – Yes 2 – No  1 – Yes 2 – No  1 – Yes 2 – No  1 – Yes 2 – No  1 – Yes 2 – No |
| 36.In your family, someone has/had a problemto get intoxicated (alcohol abuse)?Embryo | | | | | | Yes 1  Not 2 |
| 37.If yes, who hashad thisalcohol abuse problem?  VC37 Yourself  ES 37 Spouse/Companion  FI37 son/daughter  *(Kinship in relation to the child)*MP37Mother/Father  TI37 Tio/TIA  IR37 brother/Sister  OUT37 OU37Q Other: _________________ ____________________...... | | | | | | 1 – Yes 2 – No  1 – Yes 2 – No  1 – Yes 2 – No  1 – Yes 2 – No  1 – Yes 2 – No  1 – Yes 2 – No  1 – Yes 2 – No |
| 38.In your family, someonehas been having trouble usingdrugs, huh?Drog | | | | | | 1- Yes 2 - No 3-do not know 4-Can not ask |
| 39.If yes, Quandtype of drughave you been using? MA39Marijuana  CO39 Cocaine  CR39 Crack  CL39 Cola  CP39 tablets  OUT39 OU39Q Other: ________________________________ ...... | | | | | | 1 – Yes 2 – No 3-do not know  1 – Yes 2 – No 3-do not know  1 – Yes 2 – No 3-do not know  1 – Yes 2 – No 3-do not know  1 – Yes 2 – No 3-do not know  1 – Yes 2 – No 3-do not know |

Interorganizer: _______________________________________________________________________________________________________________________________________________________________________________________________________________________________________________________________________________________________________________________/ ___/_____

| UFC/ Unichristus/FUNCAP-CNPq  **VI Maternal-infant Health survey in CEARÁ-2017** | WOMAN'S INFORMATION10-49 YEARS |
| --- | --- |

01. Municipality: ________________________________________ woman's name: "Women's Names" __________________ Contact Phone: ____________________.................

02. Quiz: [MUN] Icipio: _____ [sector]: ________ ***(4 last Nos. MAP)*** [home]: _____ [woman]: ____

| 03. How old are you?  [IDAMUL] | Years _____ |
| --- | --- |
| 04. Skin color:  Skin  OUT04 OU04Q | White 1  Parda 2  Black 3  Other: ___________________ 4... |
| 05. Can you read and write?  READ | Yes 1  Not 2  Just sign 3 |
| 06. Until year (series) did you study at school? *(Passed from year)*Be  ***(If top level, note how many years, in ' series ', and 3rd. Degree)*** grade | ___ Series  ___ Degree |
| 07. What is your marital status?  COMPA | Maiden 1  Married 2  Stable Union 3  4 separate  Widow 5 |
| The 08th. What's your religion?  RELIG  OUT08 OU08Q | Catholic 1  Protestant/Evangelical 2  Spiritist 3  Umbanda/Candomblé 4  Other: ___________________ 5...  No 6 |
| 09. If you have religion, do you consider yourself a practicing practitioner?  PRAT | Yes 1  Not 2 |
| Ten, ten. V. Do you currently work?  *(Work with some gain or not)* [Traba] | Yes, only at home (housework) 1  Yes, out of the House 2  Yes, at home, out 3  Does not work at all (not even household) 4 |
| 11. In your work (or at home, if you don't work) V. Spends most of the time  In what position?  KNEELS  *(Read the options)*  OUT11 OU11Q | Sitting 1  Standing 2  Walking 3  Making a lot of effort 4  Other: ________________ 5.... |
| 12. How many hours a day do you work?  HOTRA | ______ hours |
| 13. How many days in week V. Do you work?  DITRA | ___ Days |
| 14. In the last 30 days, how many days missed work:  *(If not missed, note 00 (zero))*Fte for emotional problems  FTD by disease, physical problems  FTP for personal reasons | ____ Days  ____ Days  ____ Days |
| Onefive. Do you smoke cigarettes? How often?  SMOKE | Yes, every day 1  Yes, a few days 2  No, you quit smoking 3  No, you never smoked four |
| Onesix. Are you satisfied with your body, or do you think you're thin, fat?  [SATCOR] | Yes, satisfied 1  No, I'd like to lose weight 2  No, I'd like to get Fat 3  You don't know 4 |
| Oneseven. In the last 30 days, did V. Do anything to lose or gain weight?  *(Does not include work activities)* CA17Hike  AC17 Academy  ES17 Sports  DI17 Diet/regimen  RE17 took slimming medicine  OUT17 OU17Q Other: _________________ ________________________ ...... | 1-Yes 2-no  1-Yes 2-no  1-Yes 2-no  1-Yes 2-no  1-Yes 2-no  1-Yes 2-no |
| 18. Has a doctor ever told you that V. Had Diabetes?  DIABE | Yes 1  Yes, when I was pregnant 2  He said he had pre-diabetes or suspicion 3  Not 4 |
| 19. If yes, what does V. Use/do to control Diabetes?  [REDIAB  OUT19 OU19Q | Insulin, regularly 1  Insulin, not regularly 2  Oral medication, regularly 3  Oral medication, not regularly 4  Other: _____________ _ __________________________ 5 in the  Does not use/do anything 6 |
| 20. Have you ever had a doctor, or other health care professional, ever told you  That V. had Arterial hypertension (high blood pressure)? [HIPART] | Yes 1  Yes, when I was pregnant 2  Said he had BP in the limit (pre-hypertensive) 3  Not 4 |
| 21. If yes, what does V. Use/do to lower the pressure?  REHA | Oral medication, regularly 1  Oral medication, not regularly 2  Diet, reduces salt 3  Other: _______________ ____________ 4......  Does not use/does anything 5 |
| Twotwo. Did a doctor ever tell you that V. Had high cholesterol?  CHOLESTA | Yes 1  Not 2  Does not remember 3  You don't know what cholesterol is 4 |
| 23. Has a doctor ever told you that V. had thyroid problem?  THYROID | Yes 1  Not 2  Does not remember 3  Don't know what thyroid is 4 |
| 24. Have you ever had a doctor tell you that V. Had some of these problems in sight: MI24Miopia (see little by far)  HI24 Hhyperopia (see little Close)  VC24 VIsta tired  CA24 CAtarata  OUT24 OU24Q Other: ________________________________ ...... | 1-Yes 2-no  1-Yes 2-no  1-Yes 2-no  1-Yes 2-no  1-Yes 2-no |
| Twofive.V. Feel any chronic pain (which never passes; or that passes, but always comes back)? How much does she bother?  DORC | Yes, it bothers a lot 1  Yes, but it doesn't bother you very much 2  ***(If not, pula for Q31)🡪***Not 3 |
| The 26th.If so, in which place of the body is this pain stronger?  DORL | In the head 1  Back 2  In Rooms 3  Legs 4  On arms/shoulders 5  Neck 6  Other: __________________________ 7...... |
| The 27th. If yes, how long have you had this pain problem?  Dort | ____ Years |
| The 28th. Which of these problems do you usually have/feel when you're in a pain crisis?  CD28 bend over so much pain  CC28 not caring for the house/making food  FT28 missing work/school  SE28 having to search for an emergency service  AN28 having to use strong/narcotic analgesic  CA28 having to take calming (for nerves/depression)  OCT28 or28Q Other: ____________________________ ......... | 1-Yes, always 2-yes, sometimes 3-no  1-Yes, always 2-yes, sometimes 3-no  1-Yes, always 2-yes, sometimes 3 – no  1-Yes, always 2-yes, sometimes 3 – no  1-Yes, always 2-yes, sometimes 3 – no  1-Yes, always 2-yes, sometimes 3 – no  1-Yes, always 2-yes, sometimes 3 – no |
| The 29th. Has any doctor ever told you what causes this pain?  DORM  OCT29 or29Q | Problem in column 1  Problem in a nerve 2  Tumor 3  Swelling 4  Migraine 5  Other: ________________ 6....  Did not consult a physician 7 |
| Three0. What do you think causes this pain?  DORV    OUT30 OU30Q | What the doctor said 1  ' A bad way that gave ' 2  ' Evil eye, Breakanto, backrest ' 3  One fall, hit 4  One: __________________________________________________________________________________  You don't know 6 |
| Threeone.Someone from home already said that V. Ronca, and if the snoring bothers?  OINK | Yes, snore and bother 1  Yes, snore but it doesn't bother 2  Not 3 |
| Threetwo.Has Anyone in the family ever seen V. Stop breathing while you sleep?  PRESP | Yes, often 1  Yes, sometimes 2  Yes, rarely 3  Not 4 |
| 33 in the last 12 months, did you consult with doctor, nurse or dentist?  Medical CONSM  CONSE Nurse  CONSD Dentist | Yes 1 No 2  Yes 1 No 2  Yes 1 No 2 |
| Threefour. If you consulted with a doctor, what was the MOTIvo of the last appointment? withtheT  COMQ | Disease 1  Qual: ____________________________________.......  Prevention 2 |
| 35. Have you ever been vaccinated against HPV? How many doses did you receive?  ***(Note 0 If you never received; If you don't remember how many you received, write down 1)***VHPV | In. of doses ___  You don't know 9 |
| 36. Have you ever been vaccinated against rubella? How many doses did you receive?  ***(Note 0 If you never received; If you don't remember how many you received, write down 1)***Vrub | In. of doses ___  You don't know 9 |
| 37. Have you ever been vaccinated against hepatitis B? How many doses did you receive?  ***(Note 0 If you never received; If you don't remember how many you received, write down 1)***Vheb | In. of doses ___  You don't know 9 |
| 38. V. Have you vaccinated against measles?  VSAR | Yes, recently 1  Yes, as a child 2  Not 3  You don't know 4 |
| 39. Have you ever received a tetanus shot vaccine in your life? How many shots?  ***(Note 0 If you never received; If you don't remember how many you received, write down 1)***Tevida | In. of doses ___  You don't know 9 |
| 40. Have you ever taken ferrous sulfate?  Suf | Yes, received at the Health Center 1  Yes, he bought it at the pharmacy 2  Not 3  You don't know 4 |
| 41. Have you ever taken folic acid?  Acf | Yes, received at the Health Center 1  Yes, he bought it at the pharmacy 2  Not 3  You don't know 4 |
| Fourtwo. Did a doctor ever tell you that V. Had a problem in the womb?  If yes, what problem?  Uhave  OCT42 or42q | Yes, endometriosis 1  Yes, myoma (benign tumor of the uterus) 2  Yes, another tumor of the uterus 3  Not 4  Other: ______________________________ 5......... |
| 43.Have you ever done any of these surgeries?  HI43 Hysterectomy (withdrawal of the uterus)  PE43 perineum (repair vagina)  MA43 mastectomy (withdrawal of breast (s))  TI43 thyroidectomy (thyroid withdrawal) | 1-Yes 2 – Waits 3 – No  1-Yes 2 – Waits 3 – No  1-Yes 2 – Waits 3 – No  1-Yes 2 – Waits 3 – No |
| Fourfour. Have you been taking cancer prevention (gynecological) in the last 12 months?  PRECA12 | Yes 1  Not 2 |
| Fourfive. If not, have you ever done it before?  'VE PRECAUTIONS | Yes 1  Not 2 |
| Foursix. Have your breasts been examined in any consultations in the last 12 months?  *(Does not include the exam done in a prenatal consultation)* Exsin' | Yes 1  Not 2 |
| Fourseven. Have you ever had mammography exams? How many tests have you done?  ***(If you did, but don't remember how many, write down 1)*** Exmaq | Yes, ___ exams  Never did 8  You don't know what mammography is 9 |
| 48. Have you ever had an HIV test?  ***(If not or don't know, swipe to Q 51)***Exaids | Yes 1  Not 2  You don't know 3 |
| 49. If yes, why did you take this exam?  PQEXAID  *(Ler the options; Mark more than one option if appropriate)* | I wanted to know 1  The doctor asked for 2  She was pregnant 3  Donated Blood 4 |
| 50. Did you receive the resultsof this exam? At the same time (Tthis fast R) or days later? RESEXA  ***(OBS: do not ask about what was the result of the exam)*** | Yes, same time 1  Yes, he got 2  How long after: ________________________________________...  Did not receive 3 |
| 51. How many years did you have when the first rule came (menstruation)?  ***(Note 00 If you have not yet; and pass to question 54)*** Primen | Years _____ |
| 52. Which of these problems do you usually have/feel when your period comes?  ES52 Excess bleeding  CD52bend over so much pain  CC52 not caring for the house/not making food  FE52 skipping work/school  SE52 have to seek emergency service  AN52 having to use strong/narcotic analgesic  AC52 having to take contraceptive to control  CR52 having to take calming (remedy for nerves)  OCT52 or52q Other: ____________________________ ........ | 1-Yes, always 2-yes, sometimes 3-no  1-Yes, always 2-yes, sometimes 3-no  1-Yes, always 2-yes, sometimes 3-no  1-Yes, always 2-yes, sometimes 3-no  1-Yes, always 2-yes, sometimes 3 – no  1-Yes, always 2-yes, sometimes 3 – no  1-Yes, always 2-yes, sometimes 3 – no  1-Yes, always 2-yes, sometimes 3 – no  1-Yes, always 2-yes, sometimes 3 – no |
| 53. Do you usually do some intimate hygiene when you come to menstruation?  If yes, which one? Higi  OCT53 or53Q | Yes, I have water/soap 1  Yes, it uses absorbent 2  Yes, grooming and absorbent 3  Yes, another: ______________________________________ 4 ...  Doesn't do anything special 5 |
| 54. Have you ever had your first sexual intercourse? How many years did you have on the occasion?  ***(Sand has not yet had, thenote 00; 99 If not remember)***Prirel | Years _____ |
| **IF you HAVE NOT YET HAD SEXUAL INTERCOURSE, PASSand THE question 107** | |
| 55. Currently V., or your companion, use some method to avoid children?  Which method do you use most often?  METOQ | Coitus interrupted 01  Breastfeeding 02  Table 03  Temp. Basal/Billings 04  Diaphragm 05  IUD 06  Male condom 07  Female condom 08  Pill 09  Injection 10  Jelly 11  Vasectomy 12  Other: ___________________ 13.  No 14 |
| 56. If you do not use any methods, why not use?  METPQ | It's not sexually active 01  Sterility 02  Menopause 03  **Fallopian connection 04**  Vasectomy 05  Can not buy method 06  No method on unit 07  Cannot use method 08  Do not want to use method 09  Companion does not want 10  Wants to get pregnant 11  She's pregnant 12  Other: ________________ 13... |
| 57. Have you had any sexual intercourse in the last 30 days?  The RELSEX | Yes 1  Not 2  I didn't want to answer three |
| 58. If yes, was the condom used in the last sexual intercourse?  Camus | Yes, male condom 1  Yes, female condom 2  Didn't use 3 |
| 59. If not, why didn't you use the condom?  CAMNUS | Do not like to use 1  I didn't want to use two  Partner didn't want to use 3  Trust Partner 4  You can't buy 5  Already uses another method 6  Others: ____________________ 7.... |
| 60. Have you ever heard of, or have you used, emergency contraception (' next day pill ')? ANTIEMETIC  *(used shortly after the relationship, when there is a risk of becoming pregnant)* | Yes, you've heard 1  Yes, you've used two  Not 3 |
| 61. Have you ever been pregnant? Pregnant  *(consider gestation that ended in abortion)* | Yes 1  Not 2 |
| **If you NEVER got PREGNANT, you'LL SEE question 107 (Mental Health (depression))** | |
| 62. How many times have you been pregnant?  *(including Gstations that ended in abort)*Gravez | Gravidezes _____ |
| 63. At what age did you get pregnant for the first time?  The GRAPRIM | Age _____ |
| 64. At what age did you have your first child?  FILPRIM | First child _____ |
| 65. How many children have you had? (does not include abortions)  FILTI | Children taken _____ |
| 66. Of these, how many were born dead?  *(from 7 months (or 28 weeks) of gestation)* Natim | Dead children _____ |
| 67. And how many were born alive?  NATIV | Living Children _____ |
| 68. Of the children who were born alive, how many are alive to this day?  Alive | Living Today _____ |
| 69. And how many died?  Dead | They died ___ |

| 70. Of the children who were born alive, how many were born in **May Dand 2014** here?  NASC14 | | | Children _____ |
| --- | --- | --- | --- |
| 71. Of the children who **died**: | | | |
| 71a. What is thebirth? | 71B. How old were you when you died? | 71C. That he died (cause of death)? | |
| U71 last ___/ ___/_____    P71 Penul. ___/___/______  A71 tonpen. ___/ ___/_____ | U71M days ______ months  P71M days ______ months    A71M days ______ months | U71C _________________________________________________________________________________________________________________________________________________  P71C _____________________________________________________________________________________________________________________________________________________  A71C _________________________________________________________________________________________________________________________________________________________ | |

| Seventwo. Have you had an abortion in the last 12 months?  Abort | Yes 1  ***(If not, skip to Q76) 🡪***Not 2 | |
| --- | --- | --- |
| Seventhree. If so, how many abortions were spontaneous? Abesp  And how many were provoked? Abpro | Spontaneous ___  Provoked ___ | |
| Sevenfour. Of the spontaneous abortions, how many V. PREcisou go to a hospital? HESP  Of the abortions provoked, how many V. Did you need to go to a hospital? Hpro | Spontaneous ___  Provoked ___ | |
| Sevenfive. Which of these problems did you have at the last abortion?  AN75 Anemia  IN75 Infection  OUT75 OU75Q Other: _________________ ____________________ ...... | Yes 1 No 2  Yes 1 No 2  Yes 1 No 2 | |
| **CHECK IF YOU HAVE HAD DELIVERY IN THE LAST 3 YEARS (MAY 2014, HERE), IF IT DOES NOT GO TO QUESTION 103** | | |
| **76. What is the date of your last delivery? *(Must be equal to the date of birth in the child's questionnaire)*** | | **__ __ / __ __ / __ __ __ __** |
| Sevenseven. In Pregnancy Ddidthat last child make how many prenatal consultations?  *(No query = 00)* [Pncon] | N ª of queries _____ | |
| Seveneight. How many months of pregnancy did the first prenatal visit?  [PNPRI] | Months ___ | |
| 79. Have you taken ferrous sulfate (for anemia) in this last pregnancy?  [PNFER] | Yes 1  Not 2 | |
| Eight-ten. Have you taken folic acid in your last pregnancy?  [PNAFO] | Yes 1  Not 2 | |
| Eightone. During the prenatal period, in this last pregnancy, were your breasts examined?  [PNMAM] | Yes 1  Not 2 | |
| Eighttwo. During the prenatal period, in this last pregnancy, was gynecological (internal) taken? [Pngin] | Yes 1  Not 2 | |
| Eightthree. Do you have a notebook or a pregnant woman's card? Can you show it?  CARD | Yes, seen 1  Yes, not seen 2  You don't have 3 | |
| 84. If you show the Passbook, note the No. Of the SUS card:  ***(In "identification", beginning of the new booklet)*** Carsus | __ __ __ __ __ __ __ __ __ __ __ __ __ __ __ | |
| 85. What was the probable date of childbirth?  ***(If there is no CaDerneta, annotate date informed by the mother)***Dpp | DPP _____/____/________ | |
| 86. What was the date of the birth?  ***(If you do not have a passbook, annotate your mother's date)***Dpa | DP _____/____/________ | |
| 87. How many consultations were made by trimesters of pregnancy?  CARTRI  ***(If you don't have Caderneta, annotate*** Mother's ***information )*** | 1st Trim (1-12 sem.) ___  2nd. Trim (13-24 sem.) ___  3rd Trim (25 or + without.) ___ | |
| 88. Are there weights noted on the card?  CARPES  *(Note and note)* | Yes, in all queries 1  Yes, in some queries 2  Not 3 | |
| 89. Are there blood pressure measurements noted on the card?  CARP  *(Note and note)* | Yes, in all queries 1  Yes, in some queries 2  Not 3 | |
| 90. Did you do what types of exams during the prenatal period of this last child?  PNSAN Blood Test  ***(Read the options)*** [Pnuri]Urine Test  *(****See card, if any)***[PNVDRL]VDRL Exam (syphilis)  [PNHIV] HIV Test (Aids)  [PNULS] Ultrasound Examination | 1-Yes 2-No 3-do not know  1-Yes 2-No 3-do not know  1-Yes 2-No 3-do not know  1-Yes 2-No 3-do not know  1-Yes 2-No 3-do not know | |
| 91. Have you received the tetanus shot vaccine in this last pregnancy? How many shots?  PNTET  ***(Note 0 If you have not received the vaccine)***  *(****See card, if any)*** | Yes, No. of doses ___  No, I had already received 7  No, never received 8  You don't know 9 | |
| 92. In the prenatal period V. received some guidance, or referral,  About the hospital where you should have your birth? Parori | Yes, it was oriented 1  Yes, it was forwarded 2  Not 3 | |
| Ninethree. Where was the birth?  PAROND, Paris  QMUN | City Hospital 1  Hosp. From another municipality 2  Which Mun? _____________ In house 3-_______________ | |
| Ninefour. Who attended the delivery?  LONGTO | Doctor 1  Nurse 2  Midwife 3  Other: _______________ ____________ 4...... | |
| Ninefive. How was the birth?  CUSTOMIZER | Normal 1  Forceps 2  Caesarea (1st time you did a cesarean) 3  Caesarea (had already done another cesarean) 4 | |
| Ninesix. If Caesarea, was the surgery urgent or with a marked day?  Cesa | **Urgency** (Had already entered into labor) 1  **Urgency** (had Not yet entered into labor) 2  Caesarea withmarked D ia 3 | |
| Nineseven. If it was a marked day, who decided that the birth would be Caesarea?  DCESA  OU97Q | The physician suggested 1  The Doctor has decided 2  You asked for 3  You and the doctor have decided 4  Other: _____________ __________________________ 5...... | |
| Nineeight. Did the child breastfeed in the chest right after the birth?  *(still in the delivery room)* Mapito | Yes 1  Not 2 | |

| 99. Did you have any of these health problems after childbirth?  POSDOR Headache  POSMAM inflamed breasts  POSFEB Fever  POSEC secretion with bad smell  FISTU Fistula (Bladder passage-vagina)  POSIU Urinary Tract infection  POSPA High Pressure  POSCON Seizure/attack  POSAMA Breastfeeding problem  POSAN Bleeding  POSOUT Posouq Other: ___________________________ _ ____________________....... | 1-Yes 2-no  1-Yes 2-no  1-Yes 2-no  1-Yes 2-no  1-Yes 2-no  1-Yes 2-no  1-Yes 2-no  1-Yes 2-no  1-Yes 2-no  1-Yes 2-no  1-Yes 2-no |
| --- | --- |
| 100. Did you consult the birth review during the guard?  ***(Within 45 days after delivery)*** [Poscon] | Yes 1  Not 2 |
| 101. Were you visited at home by someone from health in the first 30 days after childbirth?  [POSDIAS] | Yes, health Agent 1  Yes, physician or nurse of the PSF 2  Not 3 |
| 102. During pregnancy, in childbirth or in the guard, did you receive any guidance on how to breastfeed the child? Origes in gestation  ORIPAR in Childbirth  Oripos in the guard | 1-Yes 2-no  1-Yes 2-no  1-Yes 2-no |
| **Questions 103 to 106 only for women with Trompas connection (Q56-04). IF NOT, SWIPE TO Q. 107** | |
| 103. How many years did you have when you made the call?  [LIGANOS] | Years ______ |
| 104. How many living children did you have when you made the call?  [LIGAFIL] | Children ______ |
| 105. How was the connection made?  [LIGAPAR] | In caesarean section 1  Postpartum (shortly after delivery)2  In the interval (some time after childbirth)3 |
| 106. Who made the decision to make the call?  [LIGADEC] | Woman herself 1  The couple 2  The companion 3  Doctor 4 |
|  | |
| **107. Questions on Mental Health (depression) – SRQ20** | |
| 107.1.Do You have frequent headaches?  DC107 | Yes 1  Not 2 |
| 107. 2.Lack of appetite?  FA107 | Yes 1  Not 2 |
| 107. Do you sleep badly?  DM107 | Yes 1  Not 2 |
| 107.4. Do You scare easily?  AF107 | Yes 1  Not 2 |
| 107.5. Do You have shaking hands?  TM107 | Yes 1  Not 2 |
| 107.6. Do You feel nervous, tense, or worried?  NT107 | Yes 1  Not 2 |
| 107.7.Do You have bad digestion?  MD107 | Yes 1  Not 2 |
| 107.8.Are You Having trouble thinking clearly?  DP107 | Yes 1  Not 2 |
| 107.9.Have you been feeling sad lately?  ST107 | Yes 1  Not 2 |
| 107.10.Have You been crying more than usual?  CH107 | Yes 1  Not 2 |
| 107.11.Find difficulties to accomplish with satisfaction your daily activities?  SA107 | Yes 1  Not 2 |
| 107.12.Are You Having trouble making decisions?  TD107 | Yes 1  Not 2 |
| 107.13.Has difficulties in the service (his work is painful, causes suffering)?  DS107 | Yes 1  Not 2 |
| 107.14.Are you incapable of playing a useful role in your life?  PU107 | Yes 1  Not 2 |
| 107.15.Have you lost interest in things?  PC107 | Yes 1  Not 2 |
| 107.16.Do You feel like a useless person, no use?  PI107 | Yes 1  Not 2 |
| 107.17.Have you had any idea of ending life?  AV107 | Yes 1  Not 2 |
| 107.18. Do You feel tired all the time?  CT107 | Yes 1  Not 2 |
| 107.19.Do you get tired easily?  CF107 | Yes 1  Not 2 |
| 107.20. Do you have unpleasant sensations in your stomach?  SD107 | Yes 1  Not 2 |

| **Domestic or intimate partner violence:** | |  |
| --- | --- | --- |
| 108.Do you consider yourself safe at home?  Segur | Yes 1  Not 2 |  |
| 109.Does Anyone hit you or call you names (Xinga)?  Slaughter | Yes 1  Not 2 |  |
| 110.Have you ever pushed, knocked, kicked, punched another adult?  VBATE | Yes 1  Not 2 | |
| Oneeleven.Have you ever been pushed, beaten, kicked, punched by another adult?  The OBATE | Yes 1  Not 2 | |

| 112. In the last 12 months, which of these health problems have you had?  DE112 Dengue Fever  ZI112 Zika  CH112 Chikungunya  AL112 Allergy  SI112 Sinusitis  PN112 Pneumonia  OUT112 OU112Q Other: _________________ ________________...... | 1-Yes 2-no  1-Yes 2-no  1-Yes 2-no  1-Yes 2-no  1-Yes 2-no  1-Yes 2-no  1-Yes 2-no |
| --- | --- |

| **ANTHROPOMETRICAL EXAMINATION OF WOMEN** | |
| --- | --- |
| Eleventhree. Waist circumference (CC):  *(Measured just below the ribs)* Circin | **C** C **(cm):** __________, ___ |
| Elevenfour. Abdominal circumference (CA):  *(Measured at the height of the navel)* Circab | **CA (cm): __________, ___** |
| Elevenfive. Hip circumference (CQ):  *(Measured at the gluteus height)* CIRCQD | **CQ (cm): ________, ___** |
| Elevensix. Weight (W):  PESOM | **P (kg): ________, ___** |
| Elevenseven. Height (E):  The STATM | **E (cm): ________, ___** |

Interera: ____________________________________________________________________________________________________________________________________________________________________________________________Data: ___/___/_____

| UFC/ Unichristus/FUNCAP-CNPq  **VI Maternal-infant Health survey in CEARÁ-2017** | INFORMATION FROMCHILD under 6 years old |
| --- | --- |

01. Municipality: ____________________________________________________________ Child: ________________________________________________________________________________________________________________________

02. [MUN]: _____ [Sector]: ______ ***(4 last Nos. MAP)*** [home]: _____ [woman]: ____ No.[CRI] ANÇA: ___

| 03. What is the sex of * (from here speak the name of the child)?  SEX | | Male 1  Female 2 | | |
| --- | --- | --- | --- | --- |
| **04. What is the birth date of *?**  ***(It should be equal to the last calving date (Q76) in the MulheR questionnaire,*** Datn  ***If you are the youngest child)*** | | | **Date: _____/____/________** | |
| 05. How old is *?  AGE | | Years: ___ months: _____ | | |
| 06. What is V.?  RESPOND | | Biological Mother 1  Adoptive mother 2  Grandmother 3  Tia 4  Sister 5  Other: ________________ 6...... | | |
| 07. Does the mother of * live in the house?  LIVE | | Yes, the birth mother 1  Yes, the adoptive mother 2  No mother died 3  Doesn't live 4 | | |
| 08. The father of * lives in the house?  [MORAP]  ***(If you live biological or adoptive father, skip to Q12)*** | | Yes, the biological father 1  Yes, the adoptive father 2  No, the stepfather lives 3  No father died 4  Does not live 5 | | |
| 09. If you don't live at home, does the biological father See/Stay with the child?  PAIVE | | Yes, always 1  Yes, sometimes 2  Not 3 | | |
| 10. If you don't live at home, Do you receive any help from the Child 's biological father?  PAIAJ | | Yes, alimony 1  Yes, another help 2  Not 3 | | |
| 11. If you don't live at home, would you like to see the child's biological father more often?Paifr | | Yes 1  Not 2 | | |
| 12. When you got pregnant from the last child V. Wanted to get pregnant?  WANTED TO | | Yes, I'd like 1  I didn't plan, but I was happy 2  No, I wanted another moment 3  No more son 4  I never accepted this son 5  NS/NQR 6 | | |
| 13. Which of these people care for the child at home?  MC13 mom  PC13 Pai  AC13 Grandma  TC13 Tia  IC13 Sister  OUC13 OC13Q Other: ____________________________ ...... | | 1-Yes 2-no  1-Yes 2-no  1-Yes 2 – no  1-Yes 2-no  1-Yes 2-no  1-Yes 2-no | | |
| 14. Did Mrs. (the mother) take maternity leave?How many months?  LIMAT  Limes | | Yes 1  Months: _____  Not 2 | | |
| 15. Mrs. (mother) Have you taken ferrous sulfate?  Sfm | | Yes, before pregnancy 1  Yes, during pregnancy 2  Yes, before and during pregnancy 3  You haven't taken 4  You don't know 5 | | |
| 16. Mrs. (mother) took folic acid?  Afm | | Yes, before pregnancy 1  Yes, during pregnancy 2  Yes, before and during pregnancy 3  You haven't taken 4  You don't know 5 | | |
| 17. Did Mrs. (the mother) smoke during the child's gestation?  Fum | | Yes, smoked a little bit 1  Yes, smoked moderate 2  Yes, he smoked a lot 3  Did not smoke 4 | | |
| 18. Mrs. (mother) drank during the child's gestation?  Well  *(Alcoholic beverages)* | | Yes, he drank a little bit 1  Yes, he drank moderate 2  Yes, he drank a lot 3  Didn't drink 4 | | |
| 19. Does the child live in another house besides this?  Listen | | Yes 1  Not 2 | | |
| 20. IF Yes, whose other house is it?  OUCAQ  OUC20 OC20Q | | From Mother 1  From Father 2  Grandparents 3  From Aunt/Uncle 4  From the Godmother/Godfather 5  Other: ________________ 6...... | | |
| The 21st. Does * have a declaration or birth certificate?  [DECLNAS] | | Yes, statement 1  Yes, certificate 2  Not 3 | | |
| 22. Does * have the child's booklet?  CARD  *(Ask to see the Caderneta)* | | Yes, seen 1  Yes, not seen 2  No, you lost 3  Never had 4 | | |
| **Information 22a. and 22b. To be copied from the child'S booklet (see page 39)** | | | | |
| 22a. Birth Data:  Birth weight: ________ g length at birth: _______ cm cephalic circumference: ____ , __cm*(note 00 if not filled)*  **Apgar:**1st min: _____ 5th min: ____ Gestational age (IG): _____ Weeks _____ days | | | | |
| 22b. Screening Tests:  Ortolani Maneuver  Reflexo VEd test  Pezinho Test  Screening theuditiva | | 1- Negative 2 - positive3-not filled  1- Normal2- changed3-not filled  1- N 2-yes 3-not filled  1-No 2-yes 3-not filled | | |
| 23. How much did the child weigh at birth? *(Ignored = 9999)*  OVERAT  ***(Ask mother if weight is not noted in Cardeneta)*** | | Birth weight confirmed in the booklet: ____________ g  Birth weight informed by the mother: ____________ G | | |
| 24. Was the pregnancy only of this child, or was it twins?  TWIN | | Simple Pregnancy 1  Twin Pregnancy 2 | | |
| 25. The * was born of time, before time or after time?  [NASTEMP] | | Born of Time 1  Born before Time (premature) 2  Born after Time 3  You don't know 4 | | |
| 25a. In what position (in the belly) was the baby at the time of birth?  Position | | Baby was head 1  Baby was sitting (buttocks) 2  The baby was traversed 3  You don't know 4 | | |
| Twosix.Thebaby was hospitalized after childbirth?  RNINT | | Yes, in a nursery 1  Yes, in an incubator 2  Yes, in an ICU 3  No, she was discharged with her mother 4 | | |
| Twoseven. If yes, how many days have you been hospitalized?  RNINQ | | ______ Days | | |
| Twoeight. If yes, the baby needed:  RE28 Resuscitation  OX28 Oxygen  AB28 Antibiotics  AS28 feeding by probe  BL28 Bath Light  CG28 be warmed by the mother (kangaroo) | | 1-Yes 2-No 3-do not know  1-Yes 2-No 3-do not know  1-Yes 2-No 3-do not know  1-Yes 2-No 3-do not know  1-Yes 2-No 3-do not know  1-Yes 2-No 3-do not know | | |
| 29. Did the child breastfeed in the first hour after birth?  MAMPH | | Yes 1  Not 2 | | |
| 30. O * Breast in breast?  BREAST | | Yes 1  Not 2 | | |
| 31. If not, how old did * breast breastfeed? MAMOU  *(age in months)* | | Months ______  Never sucked 77 | | |
| 32. At what age * began to receive: *(age in months)* [Outalim]    ***(Quote options)*** | | Water or tea: ______  Other milk: ______  Mingau (milk + pasta): ______  1 solid foods: ______  Cooking Pot: ______ | | |
| 33. How many doses of these vaccines has the child taken? Bcg  HEPB  **(*Check the child's booklet, page 82 (old), page 84 (new))***Sabin  Penta  *(Add and write down the No. Doses of each vaccine)* Rotav  PNEUMATIC  *(Note 0 (zero) if you have not taken any dose)* Menin  FARA  Hepa  *(If the mother says she took, but does not remember how many doses, note 1)*Trivir  Tetra  OUTV | | BCG (scar on ARM) ___  Hepatitis B ___  Anti-Polio (VIP/VOP) Sabin ___  Pentavalent ___  Rotavírus ___  Pneumocococcal _____  Meningococcal C ___  Yellow Fever ___  Hepatitis A ___  Viral Threefold ___  Tetra Viral ___  Another vaccine: ______________...... | | |
| 34. Source of vaccine information: [FONTV] | | 1-Passbook 2-Mother 3-both | | |
| 35. Have you ever had a dose of vitamin A?  ***(Vitamin A Capsules)*** [VITA] | | Yes 1  Not 2 | | |
| 36. If yes, how many doses (capsules) did he take?  **(*Check the child's booklet, page 81 (old), page 83 (new))***DOVA | | Servings: ____ | | |
| 37. If yes, when did he take these doses?  DOVAQ  **(*Check the child's booklet, page 81 (old), page 83 (new))*** | First. Dosage: _____/_____/________  Second. Dosage: _____/_____/________  Third. Dose: _____/_____/________ 4th. Dosage: _____/_____/________  5th. Dosage: _____/_____/________ | | | 6th. Dosage: _____/_____/________  7th. Dosage: _____/_____/________  8th. Dose: _____/_____/_______ 9 ª. Dosage: _____/_____/________ |
| 38. Has it been heavy in the last 3 months?  [PESOCAR] | | Yes, registered card 1  Yes, not registered 2  It was not heavy 3 | | |
| 39. A * has any problem of birth (congenital)? What? PCONG  PCONQ | | Yes 1  What? ______________________________________  Not 2 | | |
| 40. Has the child had any surgery? What? SCISSORS  CIRUQ | | Yes 1  What? ______________________________________  Not 2 | | |
| 41. Has the child used antibiotics in the last six months? What? The ANTBI  ANTBQ | | Yes 1  What? ______________________________________  Not 2 | | |
| 42. Has diarrhea in the last 24 hours?  [DIAR24] | | Yes 1  Not 2 | | |
| 43. Has diarrhea in the last 15 days?  [DIAR15] | | Yes 1  Not 2 | | |
| 44. Did you give any serum to treat diarrhea?  *(Oral rehydrating serum)* [Serum] | | Yes 1  Not 2 | | |
| 45. Has cough in the last 15 days? [TOS15] | | Yes 1  Not 2 | | |
| 46. If you had a cough, did you have difficulty breathing? [DIFRES] | | Yes 1  Not 2 | | |
| 47. Why did you have trouble breathing?  The CAURES  OUC47 OC47Q | | Stuffy Nose 1  Fatigue 2  Other: ________________ 3..... | | |
| 48. A * had fever? [Fever]  *(In the last 15 days when he had cough)* | | Yes 1  Not 2 | | |
| 49. Have you ever had a doctor in your life said you had asthma? ASTHMA | | Yes 1  Not 2  You don't know 3 | | |
| 50. Have you ever had any of these accidents? What's the gravity?  QE50 Fall  QM50 Burn  EO50 Swallow Objects  TR50 taking remedy/Poison  SU50 suffocation  AF50 Drowning  EC50 Electric shock  AT50 Traffic Accident  OUC47 OC47Q Other: _________________ ________________________...... | | 1-Yes, record 2-yes, moderate/light 3-No  1-Yes, record 2-yes, moderate/light 3-No  1-Yes, record 2-yes, moderate/light 3-No  1-Yes, record 2-yes, moderate/light 3-No  1-Yes, record 2-yes, moderate/light 3-No  1-Yes, record 2-yes, moderate/light 3-No  1-Yes, record 2-yes, moderate/light 3-No  1-Yes, record 2-yes, moderate/light 3-No  1-Yes, record 2-yes, moderate/light 3-No | | |
| 51. If you had an accident that you considered serious, how did you treat this accident?  [ACTRAT] | | Treated at home 1  Consulted Child 2  Hospitalized Child 3 | | |
| 52. In the last 3 months * Did you do how many appointments with a doctor? COMED  *(Check 0 If you have not made any queries)* | | ___ Queries | | |
| 53. What is the reason for the last consultation of * with the physician? MEDIUMS | | Diarrhea 1  Respiratory Infections 2  Skin Problems 3  Other: ________________ 4.....  Prevention: ________________ 5... | | |
| 54. In the last 3 months to * have you consulted at the pharmacy, with prayers or with health agents? COFARM Pharmacy  COREZA Rezadeira COAGEN Health Agent | | 1-Yes 2-no  1-Yes 2-no  1-Yes 2-no | | |
| 55 in the last 3 months a * had consultations with:  FISIO Physiotherapist  NUTRIC Nutritionist  TEROC Occupational Therapist  FONOA Speech therapist PSICOL psychologist | | 1-Yes 2-no  1-Yes 2-no  1-Yes 2 – no  1-Yes 2-no  1-Yes 2-no | | |
| 56. A * has been admitted to hospital in the last 12 months?  *(Check 0 If you have not committed)* [Internal] | | Admissions _____ | | |
| 57. If yes, how many times has been admitted by:  QUINTER  *(Check 0 If you have not been admitted for these reasons)*  OUC57 OC57Q | | Pneumonia _____  Diarrhea _____  Dengue _____  Zika _____  Chikungunya _____  Abscess _____  Other reason: _____________ ____________.......... | | |
| 58. Are you currently going to a daycare? Free or paid? DAYCARE | | Yes, public daycare 1  Yes, private daycare 2  Not 3 | | |
| 59. Are you currently going to a school? Free or paid? ESCO | | Yes, public school 1  Yes, private school 2  Not 3 | | |
| 60. If YES, how many hours a day does * stay in school or daycare?  HESC | | Hours _____ | | |
| 61. How many hours a day does * stay watching TV?  Htv | | Hours _____ | | |
| 62. How many hours a day does * stay on the Internet?  Hin | | Hours _____ | | |
| 63. How many hours a day does * stay in electronic touch devices on the screen?  Hae | | Hours _____ | | |
| 64. How many hours a day does * stay in electronic **games** (computer/Mobile/ video game)? HJE | | Hours _____ | | |
| 65. How many hours a day does * keep playing alone, no electronic gadgets?  Hbs | | Hours _____ | | |
| 66. How many hours a day does * keep playing with other children, without electronic ap.?  Hbc | | Hours _____ | | |
| 67. How many days a week does * have sports activities?  Hae | | Days _____ | | |
| 68. Which of these things does * possess?  CE68 Mobile Phone  CD68 computer (desktop or notebook)  TQ68 TV in the room  BI68 Bike  Facebook FB68  OUC68 OC68Q participates in other social networks: ________________________... | | 1-Yes 2-no  1-Yes 2-no  1-Yes 2 – no  1-Yes 2-no  1-Yes 2 – no  1-Yes 2 – no | | |
| 69. Have you ever heard of autism, a problem affecting young children?  ATRIUM | | Yes 1  Not 2 | | |
| 70. Has any doctor ever told you that the * has autism?  The AUTIM | | Yes, it does treatment 1  Yes, but no treatment 2  Not 3 | | |
| **Questions 71.1 to 71.23 on autism, then, are only for children from 15 to 30 months of age.** | | | | |
| 71.1. Does your son like to swing, to jump on his knee, lap?  BP71 | | Yes 1  Not 2 | | |
| 71.2. Does your son have any interest in other children?  IC71 | | Yes 1  Not 2 | | |
| 71. Does your son like to climb things like ladders or furniture?  SC71 | | Yes 1  Not 2 | | |
| 71.4. Does your son like to play hide and show his face or hide-and-seek?  BE71 | | Yes 1  Not 2 | | |
| 71.5. Did your son ever play make-believe, like pretend you're talking on the phone, that you're taking care of the doll, or another make-believe joke? FC71 | | Yes 1  Not 2 | | |
| 71.6. Did your son ever use his index finger to point, to ask for anything?  DP71 | | Yes 1  Not 2 | | |
| 71.7. Did your son ever use his index finger to point, to indicate interest in something?DI71 | | Yes 1  Not 2 | | |
| 71.8. Does your child know how to play with small toys (e.g. cars or blocks), without just putting it in the mouth, stirring the toy or letting the toy fall?BD71 | | Yes 1  Not 2 | | |
| 71.9. Has your child ever brought objects for you (parents) to show you this object?TO71 | | Yes 1  Not 2 | | |
| 71.10. Does your son look at you in the eye for more than a second or two?  OO71 | | Yes 1  Not 2 | | |
| 71.11. Did your son already seem very sensitive to noise (e.g. by covering his ears)?  MS71 | | Yes 1  Not 2 | | |
| 71.12. Does your son smile in response to your face or your smile?  SR71 | | Yes 1  Not 2 | | |
| 71.13. Does your son imitate you? (e.g. do you make expressions/grimaces and your child mimics?)  IV71 | | Yes 1  Not 2 | | |
| 71.14. Does your son answer when you call him by name?  RN71 | | Yes 1  Not 2 | | |
| 71.15. If you point a toy on the other side of the room, does your son look at it?OB71 | | Yes 1  Not 2 | | |
| 71.16. Does your son know how to walk?  SA71 | | Yes 1  Not 2 | | |
| 71.17. Does your son look at things you're looking at?  OC71 | | Yes 1  Not 2 | | |
| 71.18. Does your son make strange movements with his fingers near his face?  ME71 | | Yes 1  Not 2 | | |
| 71.19. Does your son try to attract your attention to his activity?  AA71 | | Yes 1  Not 2 | | |
| 71.20. Have you ever wondered if your son is deaf?  PS71 | | Yes 1  Not 2 | | |
| 71.21. Does your son understand what people say?  PD71 | | Yes 1  Not 2 | | |
| 71.22. Does your son sometimes get airborne, "looking at nothing" or walking without definite direction?ON71 | | Yes 1  Not 2 | | |
| 71.23. Does your son look at your face to check your reaction when you see something strange?OR71 | | Yes 1  Not 2 | | |

| **Family Skills for Child development** | |
| --- | --- |
| **Psychosocial stimulation:** | |
| 72. How often do people in the house, including children, have lunch or dinner together? Alju | 1 – Always 2 – sometimes 3-never |
| 73. Does your House have magazines, books or newspapers that the child can see?  RELJ | Yes 1  Not 2 |
| 74. Does the child have any children's books or pictures or drawings?  LYMP | Yes 1  Not 2 |
| 75. Every day, do you or anyone else in your family usually play, read, or chat with only the child? Famb | Yes 1  Not 2 |
| 76. Children play with many different things. I'll say some of these things or toys and would like you to tell me if you have any at home for the child to play:  BR76 Toys (ball, doll, stuffedanimal, spinning top, kite etc.)  . OC76House objects (pots, spoons, mugs etc.)  OF76 objects and materials from outside the house (stones, kindling etc.)  BT76 toys that play music  BE76 Toys fit, assemble  BM76 Motion toys, such as skipping rope, rackets, rattle, Hula hoop  LG76 Pencil, chalk, brush, colored pen for writing/drawing | 1-Yes 2-no  1-Yes 2-no  1-Yes 2-no  1-Yes 2 – no  1-Yes 2-no  1-Yes 2-no  1-Yes 2-no |
| 77. Does the child often play or play with other children?  EARRI | Yes 1  Not 2 |
| 78. Does the child have contact with family pets?  Anim | Yes, with a dog 1  Yes, with Cat 2  Yes, with Cat and dog 3  Yes, with another animal: _____________________ 4  Family does not create pet 5 |
| 79. In the last week you or the father of the child:  PB79 Toyed with her  PC79 talked to her  PD79 Took Care of her  PP79 walked with her  PA79 helped/fed her | 1-Yes 2 – no  1-Yes 2 – no  1-Yes 2 – no  1-Yes 2 – no  1-Yes 2-no |
| **Only for children under 1 year of age:** | |
| 80 in the last 3 days, you. or another person of your family over 15 years old, did any of these things with the child:  BR80 Toyed with toys that make sounds, noises, play songs  CA80 sang for the child  JB80 played ball or objects rolling  BP80 Toyed with small toys | 1-Yes 2 – no  1-Yes 2 – no  1-Yes 2 – no  1-Yes 2 – no |
| **Only for children aged 1 to 2 years:** | |
| 81 in the last 3 days, you. or another person of your family over 15 years old, did any of these things with the child:  CA81 sang  JB81 played Ball  BP81 Toyed with small toys  DP81 designed/Painted  PA81 took the child for a stroll | 1-Yes 2 – no  1-Yes 2 – no  1-Yes 2 – no  1-Yes 2 – no  1-Yes 2 – no |
| **Only for children aged 3 to 4 years:** | |
| 82 in the last 3 days, you. or another person in your family with more than 15 years of  Age, did any of these things with the child: CO82 ran with the child  CA82 sang for the child  JB82 played Ball  BP82 Toyed with small toys  DP82 designed/Painted  PA82 took the child for a stroll  QB82 Joked with Puzzle | 1-Yes 2 – no  1-Yes 2 – no  1-Yes 2 – no  1-Yes 2 – no  1-Yes 2 – no  1-Yes 2 – no  1-Yes 2 – no |
| **Only for children aged 4 to 5 years:** | |
| 83. You or another person in your family, do any of these things with the child:  EA83 teaches alphabet (Letters)  EL83 teaches reading or writing  ES83 teaches the child about how to go out with other children  EC83 teaches the child about how to behave in school  AB83 rides bicycle, horse etc. | 1-Yes 2 – no  1-Yes 2 – no  1-Yes 2 – no  1-Yes 2 – no  1-Yes 2 – no |

| **Behavior Management (questions for all children):** | |
| --- | --- |
| Eightfour. When you get out of the house, who usually takes care of the kid:  QSAI | Child's grandmother/grandfather 1  Child Father 2  Child's aunt/Uncle 3  Child < 10 years 4  Child > 10 Years 5  Neighbors 6  Go along 7  Stands alone 8  Other: __________________________ 9...... |
| Eightfive. What are you. Does when the child does not behave well, that is, does something that you. Don't you?  RE85 rebude the child  CA85 gives a punishment  SF85 makes threats  BA85 beats her  EX85 says no and explains why she should not behave like this  PA85 gives a spanking  GR85 shouts with the child  DN85 says ' no ' or ' stop '  DI85 distracts the child  SE85 Send the child to sit, go to the room, or take her away if she is not at home | 1-Yes, spontaneous 2-yes, induced 3-No  1-Yes, spontaneous 2-yes, induced 3-No  1-Yes, spontaneous 2-yes, induced 3-No  1-Yes, spontaneous 2-yes, induced 3-No  1-Yes, spontaneous 2-yes, induced 3-No  1-Yes, spontaneous 2-yes, induced 3-No  1-Yes, spontaneous 2-yes, induced 3-No  1-Yes, spontaneous 2-yes, induced 3-No  1-Yes, spontaneous 2-yes, induced 3-No  1-Yes, spontaneous 2-yes, induced 3-No |
| Eightsix. Which of these situations happen to the child?  DO86 child sleeps in the same bed as parents  FO86 the place where the child sleeps is close to the stove, sail etc.  CH86 the place where the child sleeps is close to the ground  TO86 electrical outlets are uncapped  OB86 small objects are within reach of the child  SO86 The child is alone  FC86 Let the child go out of the house  PA86 The cookware handles are out of the stove  ME86 medicines/remedies are within the reach of the child  DE86 detergent, poison, bleach are high, away from the child  SF86 knives and scissors are stored/far from the reach of the child  BF86 children can play with fireworks and campfires | 1-Yes 2 – no  1-Yes 2 – no  1-Yes 2 – no  1-Yes 2 – no  1-Yes 2 – no  1-Yes 2 – no  1-Yes 2 – no  1-Yes 2 – no  1-Yes 2 – no  1-Yes 2 – no  1-Yes 2 – no  1-Yes 2 – no |

| **(FOLLOWING QUESTIONS WITH ASQ3 QUESTIONS)** |
| --- |

| **ANTHROPOMETRIC EXAMINATION OF THE CHILD** | |
| --- | --- |
| Eightseven. Mother's weight **with** THE CHILD: [Peswto] | P (kg): ________, ___ |
| Eighteight. Weight of the mother **without** the child: [PESOM1] | P (kg): ________, ___ |
| Eightnine. Stature (E): [ESTATC] | E (cm): ________, ___ |
| NineZero. The child was measured: [POSICAO] | 1-standing 2-lying down |
| Nineone. Cephalic perimeter: [PERICEF] | PC (cm): ______, ___ |

Interera: ____________________________________________________________________________________________________________________________________________________________________________________________________________Data: ___/___/___
