# Supplementary material for: Prevalence and socioeconomic determinants of development delay among children in Ceará, Brazil: A population-based study
Source: PLoS One. 2019 Nov 5;14(11):e0215343. doi: 10.1371/journal.pone.0215343 (PMC6830766; doi:10.1371/journal.pone.0215343)
Supplement: S2 Chart — (DOC) [file pone.0215343.s002.doc]

| UFC / UNICHRISTUS / SMS / SESA / FUNCAP-CNPq  **VI PESQUISA DE SAÚDE MATERNO-INFANTIL NO CEARÁ - 2017** | INFORMAÇÕES DO DOMICÍLIO |
| --- | --- |

| 01. Município [MUN]: ________________________________________ | | | | | 02. Distrito/Bairro [DISBA]: ______________________ | |
| --- | --- | --- | --- | --- | --- | --- |
| 03. Código Município: [COMUN]: __ __ | | | | | 04. Setor IBGE [SETOR]: __ __ __ __ *(****4 Últimos Nos. do Mapa)*** | |
| 05. Zona do Setor [ZONA]: 1 - Rural 2 – Urbana | | | | | 06. Casa No. [CASNO]: ___ ___ ***(No. da sequência do Setor)*** | |
| Endereço: | Rua/Av. ____________________________________________________ No. __________ Bairro: _________________________ | | | | | |
|  | | | | | | |
| 07. Respondente do questionário: RESPON | | | | | | Mãe/esposa 1  Pai/esposo 2  Avô/avó 3  Filho(a) 4  Outro: __________________________ 5 |
| 08. Ao todo, quantas pessoas moram na casa? [MORA] | | | | | | Pessoas: __ __ |
| 09. Quantas mulheres de 10 a 49 anos de idade moram na casa? [MUAN] | | | | | | Mulheres: ___ |
| 10. Quantas crianças com menos de 6 anos moram na casa? [CRIAN]  *(crianças com até 5 anos, 11 meses e 29 dias)* | | | | | | Crianças: ___ |
| 11. Quem é o chefe da família? [CHEF]  ***(Em caso de dúvida, marcar o(a) que mais contribui com a Renda Familiar)*** | | | | | | O pai 1  A mãe 2  O avô/Avó 3  Filho(a) 4  Outro: _______________________________ 5 |
| 12. Até que ano (série) o(a) Chefe da Família estudou na escola? *(Passou de ano)*  [SER]  ***(Se tem nível superior, anotar quantos anos, em ‘série’, e 3º. Grau)*** [GRAU] | | | | | | Série ___  Grau ___ |
| 13. A família possui quantos destes bens em casa? BA13  ED13  ***(Se Não tem, anotar 0 (zero).***  AU13  MC13  LL13  GE13  FZ13  LR13  DV13  MO13  MT13  SR13 | | | | | | Banheiros ___  Empregados domésticos ___  Automóveis ___  Microcomputador ___  Lava Louças ___  Geladeira ___  Freezer ___  Lava Roupa ___  DVD ___  Micro-Ondas ___  Motocicleta ___  Secadora de Roupas ___ |
| 14. Quais destes recursos (de informática) a Família tem em casa?  Internet [IN14]  Notebook [NB14]  Tablet [TA14]  Celular (só fone) [CE14]  Smartphone (celular com toques na tela) [SM14] | | | | | | 1 – Sim 2 – Não  1 – Sim 2 – Não  1 – Sim 2 – Não  1 – Sim 2 – Não  1 – Sim 2 – Não |
| 15. De onde vem a água usada para beber? BEBER | | | | | | Encanada, dentro de casa 1  Encanada, fora de casa 2  Cisterna 3  Chafariz 4  Cacimba 5  Rio/açude/lagoa 6  Água mineral 7  Outro: ______________________________ 8 |
| 16. Qual o tipo de privada da casa? [PRIV] | | | | | | Sanitário com descarga 1  Sanitário sem descarga 2  Casinha com fossa 3  Buraco cercado 4  Não tem 5 |
| 17. Qual o tipo de pavimento da rua onde mora a família? PAV | | | | | | Asfalto 1  Calçamento 2  Terra 3  Outro: _____________________ 4 |
| 18. A família participa do Programa Bolsa Família? [BOLSA] | | | | | | Sim, recebe a bolsa 1  Sim, se cadastrou, mas não recebe a bolsa 2  ***(Se Não, pular para Q20) 🡪*** Não 3 |
| 19. Se Sim, quanto recebeu do Bolsa família no último mês? BOLSQ | | | | | | R$ __ __ __, 00 |
| 20. **Ao todo**, quanto as pessoas da família ganharam no mês passado? [REND]  *(Incluindo bolsa família, pensão, aposentadoria etc.)* | | | | | | R$ __ __.__ __ __, 00 |
| 21. Alguém na família tem plano de saúde (convênio)? [CONV]  (Unimed, IPM etc.) | | | | | | Sim, pago pela empresa 1  Sim, pago pela família 2  Não 3 |
| 22. Se Sim, qual o nome do plano? CONVN  OUT22 OU22Q | | | | | | Unimed 1  Hap Vida 2  Free Life 3  GEAP 4  IPM 5  ISSEC 6  Bradesco Saúde 7  AMIL 8  Outro: ______________________________ 9 |
| 23. Se Sim, quantas pessoas da família estão cobertas pelo plano? CONVQ | | | | | | Todas as pessoas 1  Uma parte: No. de pessoas: ____ |
| 24. Da última vez que alguém da família ficou doente, com quem se consultou?  CONSQ  OU24Q | | | | | | Médico do hospital 1  Médico do Posto (Centro) de Saúde 2  Médico da UPA 3  Médico cubano 4  Médico do Plano de Saúde 5  Médico particular (pagou do próprio bolso) 6  Outro Profissional: _____________________________ 7 |
| 25. Alguém da família (que mora na casa) já procurou fazer exames sem estar sentindo nada / sem estar doente, nos últimos 12 meses (C*heck-up*)? CHECK  CHECKQ | | | | | | Sim 1  Quem? __________________________________  *(Parentesco com a criança)* 2 – Não |
| 26. V. conhece o Programa de Saúde da Família? Já foi visitada por alguém do programa? [PSF] | | | | | | Sim, família já foi visitada em casa 1  Sim, PSF na área, mas família não visitada 2  Sim, ouviu falar 3  Não conhece 4 |
| 27. Alguém da família já teve alguma destas doenças?  IAM Infarto do miocárdio (Coração)  AVC Acidente vascular cerebral (derrame, trombose)  CAN Câncer (tipo: ___________________ )  OUT27 OU27Q Outra doença séria: ______________________________ | | | | | | 1 – Sim 2 – Não 3 – Não sabe  1 – Sim 2 – Não 3 – Não sabe  1 – Sim 2 – Não 3 – Não sabe  1 – Sim 2 – Não 3 – Não sabe |
| **Questões sobre Insegurança Alimentar (EBIA) – Todas as questões são referentes aos últimos 3 meses** | | | | | | |
| 28.01. Nos últimos 3 meses, moradores tiveram preocupação de que os alimentos acabassem antes de poderem comprar ou receber mais comida? Com que frequência isto aconteceu?  IA01 | | | | Sim, quase todo dia 1  Sim, alguns dias 2  Sim, mas só 1 ou 2 dias 3  Não 4  NS/ NQR 5 | | |
| 28.02. Nos últimos 3 meses, alimentos acabaram antes que os moradores tivessem dinheiro para comprar mais comida?  Com que frequência isto aconteceu?  IA02 | | | | Sim, quase todo dia 1  Sim, alguns dias 2  Sim, mas só 1 ou 2 dias 3  Não 4  NS/ NQR 5 | | |
| 28.03. Nos últimos 3 meses, moradores ficaram sem dinheiro para ter uma alimentação saudável e variada?  Com que frequência isto aconteceu?  IA03 | | | | Sim, quase todo dia 1  Sim, alguns dias 2  Sim, mas só 1 ou 2 dias 3  Não 4  NS/ NQR 5 | | |
| 28.04. Nos últimos 3 meses, moradores comeram apenas alguns alimentos que ainda tinham, porque o dinheiro acabou?  Com que frequência isto aconteceu?  IA04 | | | | Sim, quase todo dia 1  Sim, alguns dias 2  Sim, mas só 1 ou 2 dias 3  Não 4  NS/ NQR 5 | | |
| 28.05. Nos últimos 3 meses, algum morador de 18 anos ou mais de idade diminuiu alguma vez a quantidade de alimentos nas refeições ou deixou de fazer alguma refeição porque não havia dinheiro para comprar comida?  Com que frequência isto aconteceu?  IA05 | | | | Sim, quase todo dia 1  Sim, alguns dias 2  Sim, mas só 1 ou 2 dias 3  Não 4  NS/ NQR 5 | | |
| 28.06. Nos últimos 3 meses, algum morador de 18 anos ou mais de idade alguma vez comeu menos porque não havia dinheiro para comprar comida?  Com que frequência isto aconteceu?  IA06 | | | | Sim, quase todo dia 1  Sim, alguns dias 2  Sim, mas só 1 ou 2 dias 3  Não 4  NS/ NQR 5 | | |
| 28.07. Nos últimos 3 meses, algum morador de 18 anos ou mais de idade alguma vez sentiu fome mas não comeu porque não havia dinheiro para comprar comida?  Com que frequência isto aconteceu?  IA07 | | | | Sim, quase todo dia 1  Sim, alguns dias 2  Sim, mas só 1 ou 2 dias 3  Não 4  NS/ NQR 5 | | |
| 28.08. Nos últimos 3 meses, algum morador de 18 anos ou mais de idade perdeu peso porque não comeu quantidade suficiente de comida devido à falta de dinheiro para comprar comida?  Quanto peso ele(a) perdeu?  IA08 | | | | Sim Pouco peso 1  Sim, algum peso 2  Sim, muito peso 3  Não 2  NS/ NQR 5 | | |
| 28.09. Nos últimos 3 meses, algum morador de 18 anos ou mais de idade alguma vez fez apenas uma refeição ou ficou um dia inteiro sem comer porque não havia dinheiro para comprar comida?  Com que frequência isto aconteceu?  IA09 | | | | Sim, quase todo dia 1  Sim, alguns dias 2  Sim, mas só 1 ou 2 dias 3  Não 4  NS/ NQR 5 | | |
| 28.10. Nos últimos 3 meses, algum morador **com menos de 18 anos** de idade alguma vez deixou de ter uma alimentação saudável e variada porque não havia dinheiro para comprar comida?  Com que frequência isto aconteceu?  IA10 | | | | Sim, quase todo dia 1  Sim, alguns dias 2  Sim, mas só 1 ou 2 dias 3  Não 4  NS/ NQR 5 | | |
| 28.11. Nos últimos 3 meses, algum morador **com menos de 18 anos** de idade alguma vez não comeu quantidade suficiente de comida porque não havia dinheiro para comprar comida?  Com que frequência isto aconteceu?  IA11 | | | | Sim, quase todo dia 1  Sim, alguns dias 2  Sim, mas só 1 ou 2 dias 3  Não 4  NS/ NQR 5 | | |
| 28.12. Nos últimos 3 meses, algum morador **com menos de 18 anos** de idade diminuiu a quantidade de alimentos nas refeições porque não havia dinheiro para comprar comida?  IA12 | | | | Sim, quase todo dia 1  Sim, alguns dias 2  Sim, mas só 1 ou 2 dias 3  Não 4  NS/ NQR 5 | | |
| 28.13. Nos últimos 3 meses, algum morador **com menos de 18 anos** de idade alguma vez deixou de fazer uma refeição porque não havia dinheiro para comprar comida?  Com que frequência isto aconteceu?  IA13 | | | | Sim, quase todo dia 1  Sim, alguns dias 2  Sim, mas só 1 ou 2 dias 3  Não 4  NS/ NQR 5 | | |
| 28.14. Nos últimos 3 meses, algum morador **com menos de 18 anos** de idade alguma vez sentiu fome mas não comeu porque não havia dinheiro para comprar comida?  Com que frequência isto aconteceu?  IA14 | | | | Sim, quase todo dia 1  Sim, alguns dias 2  Sim, mas só 1 ou 2 dias 3  Não 4  NS/ NQR 5 | | |
| 28.15. Nos últimos 3 meses, algum morador **com menos de 18 anos** de idade alguma vez ficou um dia inteiro sem comer porque não havia dinheiro para comprar comida?  Com que frequência isto aconteceu?  IA15 | | | | Sim, quase todo dia 1  Sim, alguns dias 2  Sim, mas só 1 ou 2 dias 3  Não 4  NS/ NQR 5 | | |
| 29. Morreu alguma pessoa na casa (da família) nos últimos 12 meses? MOR | | | Sim 1  Não 2 | | | |
| 30. Se SIM, quem foi(ram) esta(s) pessoa(s)?  *(parentesco em relação à criança)* | | P30.1 Parentesco: _______________ I30.1 Idade: __ __ C30.1 Causa: ___________________  P30.2 Parentesco: _______________ I30.2 Idade: __ __ C30.2 Causa: ___________________  P30.3 Parentesco: _______________ I30.3 Idade: __ __ C30.3 Causa: ___________________ | | | | |
| 31. A família se mudou de casa nos últimos 12 meses?  MUD | | | | | | Sim 1  Não 2 |
| 32. Se Sim, quantas vezes se mudou? | | | | | | vezes ___ |
| 33. Nos últimos 12 meses, tem havido algum destes problema na sua família?  MO33 Morte de pessoa da família ou próximo da família  AC33 Acidente ou doença grave na família  SE33 Separação ou divórcio  DI33 Disputa por custódia de filhos/ pensão alimentícia  PE33 Alguém perdeu o emprego  PR33 Alguém foi preso  AP33 Falta de apoio da família/ dos amigos  OUT33 OU33Q Outro: ______________________________________ | | | | | | 1 – Sim 2 – Não  1 – Sim 2 – Não  1 – Sim 2 – Não  1 – Sim 2 – Não  1 – Sim 2 – Não  1 – Sim 2 – Não  1 – Sim 2 – Não  1 – Sim 2 – Não |
| 34. Na sua família, houve ou tem havido brigas, discussões outro tipo de violência em casa? (violência doméstica) VIOL | | | | | | Sim 1  Não 2 |
| 35. Nos últimos 12 meses, quais destes tipos de problemas tem havido em casa?  DI35 Discussão, bate-boca, xingamento  AG35 Agressão física  EM35 Chegar embriagado, drogado  EX35 Expulsar alguém de casa  OUT35 OU35Q Outras: __________________________________ | | | | | | 1 – Sim 2 – Não  1 – Sim 2 – Não  1 – Sim 2 – Não  1 – Sim 2 – Não  1 – Sim 2 – Não |
| 36. Na sua família, alguém tem/teve problema de ficar embriagado (abuso de álcool)? EMBRI | | | | | | Sim 1  Não 2 |
| 37. Se Sim, quem tem tido esse problema com abuso de álcool?  VC37 Você mesma  ES 37 Esposo/ companheiro  FI37 Filho/ filha  *(parentesco em relação à criança)* MP37 Mãe / pai  TI37 Tio/ tia  IR37 Irmão/ Irmã  OUT37 OU37Q Outro: ____________________________ | | | | | | 1 – Sim 2 – Não  1 – Sim 2 – Não  1 – Sim 2 – Não  1 – Sim 2 – Não  1 – Sim 2 – Não  1 – Sim 2 – Não  1 – Sim 2 – Não |
| 38. Na sua família, alguém tem tido problema de usar drogas? DROG | | | | | | 1- Sim 2 - Não 3 - Não sabe 4 - Não Pode Perguntar |
| 39. Se Sim, que tipo de droga tem usado? MA39 Maconha  CO39 Cocaína  CR39 Crack  CL39 Cola  CP39 Comprimidos  OUT39 OU39Q Outra: __________________________ | | | | | | 1 – Sim 2 – Não 3 - Não sabe  1 – Sim 2 – Não 3 - Não sabe  1 – Sim 2 – Não 3 - Não sabe  1 – Sim 2 – Não 3 - Não sabe  1 – Sim 2 – Não 3 - Não sabe  1 – Sim 2 – Não 3 - Não sabe |

Entrevistadora:_____________________________________________________________________ Data: ___/ ___/ _____

| UFC / UNICHRISTUS / FUNCAP-CNPq  **VI PESQUISA DE SAÚDE MATERNO-INFANTIL NO CEARÁ - 2017** | INFORMAÇÕES DA MULHERDE 10 - 49 ANOS |
| --- | --- |

01. Município: _____________________________ Nome da mulher: ___________________________ Telefone para contato: ______________

02. Questionário: [MUN]ICIPIO: ___ ___ [SETOR]: __ __ __ __ ***(4 Últimos Nos. do Mapa)*** [CASA]: __ __ [MULHER]: __ __

| 03. Qual a sua idade?  [IDAMUL] | Anos __ __ |
| --- | --- |
| 04. Cor da pele:  PELE  OUT04 OU04Q | Branca 1  Parda 2  Negra 3  Outra: _________________ 4 |
| 05. Sabe ler e escrever?  [LERES] | Sim 1  Não 2  Só assinar 3 |
| 06. Até que ano (série) V. estudou na escola? *(Passou de ano)* SER  ***(Se nível superior, anotar quantos anos, em ‘série’, e 3º. Grau)*** GRAU | Série ___  Grau ___ |
| 07. Qual o seu estado civil?  [COMPA] | Solteira 1  Casada 2  União estável 3  Separada 4  Viúva 5 |
| 08. Qual a sua religião?  RELIG  OUT08 OU08Q | Católica 1  Protestante/evangélica 2  Espírita 3  Umbanda/Candomblé 4  Outra: _________________ 5  Nenhuma 6 |
| 09. Se tem religião, considera-se praticante?  PRAT | Sim 1  Não 2 |
| 10. V. trabalha atualmente?  *(Trabalho com algum ganho ou não)* [TRABA] | Sim, só em casa (trabalho doméstico) 1  Sim, fora de casa 2  Sim, em casa, pra fora 3  Não trabalha em nada (nem mesmo trab. doméstico) 4 |
| 11. No seu trabalho (ou em casa, se não trabalha) V. passa a maior parte do tempo  em que posição?  POSTRA  *(Leia as opções)*  OUT11 OU11Q | Sentada 1  Em pé 2  Andando 3  Fazendo muito esforço 4  Outro: __________________ 5 |
| 12. Quantas horas por dia V. trabalha?  HOTRA | ___ ___ horas |
| 13. Quantos dias na semana V. trabalha?  DITRA | ___ dias |
| 14. Nos últimos 30 dias, quantos dias faltou ao trabalho:  *(Se Não faltou, anotar 00 (zero))* FTE Por problemas emocionais  FTD Por doença, problemas físicos  FTP Por questões pessoais | __ __ dias  __ __ dias  __ __ dias |
| 15. V. fuma cigarros? Com que freqüência?  [FUMA] | Sim, todos os dias 1  Sim, alguns dias 2  Não, parou de fumar 3  Não, nunca fumou 4 |
| 16. V. está satisfeita com o seu corpo, ou se acha magra, gorda?  [SATCOR] | Sim, satisfeita 1  Não, gostaria de emagrecer 2  Não, gostaria de engordar 3  Não sabe 4 |
| 17. Nos últimos 30 dias, V. fez alguma coisa para perder ou ganhar peso?  *(Não inclui atividades do trabalho)* CA17 Caminhada  AC17 Academia  ES17 Esportes  DI17 Dieta/ regime  RE17 Tomou remédio para emagrecer  OUT17 OU17Q Outro: _____________________________ | 1-Sim 2-Não  1-Sim 2-Não  1-Sim 2-Não  1-Sim 2-Não  1-Sim 2-Não  1-Sim 2-Não |
| 18. Alguma vez um médico já lhe disse que V. tinha Diabetes?  [DIABE] | Sim 1  Sim, quando estava grávida 2  Disse que tinha pré-diabetes ou suspeita 3  Não 4 |
| 19. Se Sim, o que V. usa/faz para controlar o Diabetes?  [REDIAB  OUT19 OU19Q | Insulina, regularmente 1  Insulina, não regularmente 2  Medicação oral, regularmente 3  Medicação oral, não regularmente 4  Outro: __________________________ 5  Não usa/faz nada 6 |
| 20. Alguma vez na vida um médico, ou outro profissional de saúde, já lhe disse  que V. tinha Hipertensão Arterial (Pressão alta)? [HIPART] | Sim 1  Sim, quando estava grávida 2  Disse que tinha PA no limite (pré-hipertensa) 3  Não 4 |
| 21. Se Sim, o que V. usa/faz para baixar a pressão?  [REHA] | Medicação oral, regularmente 1  Medicação oral, não regularmente 2  Dieta, reduz sal 3  Outro: __________________________ 4  Não usa/faz nada 5 |
| 22. Alguma vez um médico já lhe disse que V. tinha Colesterol alto?  [COLEST] | Sim 1  Não 2  Não lembra 3  Não sabe o que é colesterol 4 |
| 23. Alguma vez um médico já lhe disse que V. tinha problema de Tireóide?  [TIREO] | Sim 1  Não 2  Não lembra 3  Não sabe o que é tireóide 4 |
| 24. Alguma vez um médico já lhe disse que V. tinha algum destes problemas na vista: MI24 Miopia (Vê pouco de longe)  HI24 Hipermetropia (Vê pouco de perto)  VC24 Vista cansada  CA24 Catarata  OUT24 OU24Q Outro: __________________________ | 1-Sim 2-Não  1-Sim 2-Não  1-Sim 2-Não  1-Sim 2-Não  1-Sim 2-Não |
| 25. V. sente alguma dor crônica (que nunca passa; ou que passa, mas sempre volta)? O quanto ela incomoda?  DORC | Sim, incomoda muito 1  Sim, mas não incomoda muito 2  ***(Se Não, pula para Q31) 🡪*** Não 3 |
| 26. Se sim, em que local do corpo esta dor é mais forte?  DORL | Na cabeça 1  Nas costas 2  Nos quartos 3  Nas pernas 4  Nos braços/ombros 5  No pescoço 6  Outro: _________________________ 7 |
| 27. Se Sim, há quanto tempo tem esse problema de dor?  DORT | __ __ anos |
| 28. Quais destes problemas V. costuma ter/sentir quando está numa crise de dor?  CD28 Se curvar de tanta dor  CC28 Não cuidar da casa/fazer comida  FT28 Faltar ao trabalho/escola  SE28 Ter que procurar um serviço de emergência  AN28 Ter que usar analgésico forte/narcótico  CA28 Ter que tomar calmante (para nervos/depressão)  OUT28 OU28Q Outro: _____________________________ | 1 - Sim, sempre 2 - Sim, às vezes 3 - Não  1 - Sim, sempre 2 - Sim, às vezes 3 - Não  1 - Sim, sempre 2 - Sim, às vezes 3 – Não  1 - Sim, sempre 2 - Sim, às vezes 3 – Não  1 - Sim, sempre 2 - Sim, às vezes 3 – Não  1 - Sim, sempre 2 - Sim, às vezes 3 – Não  1 - Sim, sempre 2 - Sim, às vezes 3 – Não |
| 29. Algum médico já disse o que causa essa dor?  DORM  OUT29 OU29Q | Problema na coluna 1  Problema em um nervo 2  Tumor 3  Inchaço 4  Enxaqueca 5  Outro: __________________ 6  Não consultou um médico 7 |
| 30. O que V. acha que causa esta dor?  DORV    OUT30 OU30Q | O que o médico falou 1  ‘Um mal jeito que deu’ 2  ‘Mal olhado, quebranto, encosto’ 3  Uma queda, pancada 4  Outra: _________________________________ 5  Não sabe 6 |
| 31. Alguém de casa já disse que V. ronca, e se o ronco incomoda?  RONC | Sim, ronca e incomoda 1  Sim, ronca mas não incomoda 2  Não 3 |
| 32. Alguém da família já viu V. parar de respirar enquanto dorme?  PRESP | Sim, com frequência 1  Sim, às vezes 2  Sim, raramente 3  Não 4 |
| 33. Nos últimos 12 meses, V. fez consulta com Médico, Enfermeira ou Dentista?  CONSM Médico  CONSE Enfermeira  CONSD Dentista | Sim 1 Não 2  Sim 1 Não 2  Sim 1 Não 2 |
| 34. Se fez consulta com médico, qual foi o motivo da última consulta? COMOT  COMQ | Doença 1  Qual: _____________________________  Prevenção 2 |
| 35. V. já se vacinou contra o HPV? Quantas doses recebeu?  ***(Anote 0 se nunca recebeu; se não lembra quantas recebeu, anote 1)*** VHPV | No. de doses ___  Não sabe 9 |
| 36. V. já se vacinou contra a Rubéola? Quantas doses recebeu?  ***(Anote 0 se nunca recebeu; se não lembra quantas recebeu, anote 1)*** VRUB | No. de doses ___  Não sabe 9 |
| 37. V. já se vacinou contra o Hepatite B? Quantas doses recebeu?  ***(Anote 0 se nunca recebeu; se não lembra quantas recebeu, anote 1)*** VHEB | No. de doses ___  Não sabe 9 |
| 38. V. se vacinou contra o Sarampo?  VSAR | Sim, recentemente 1  Sim, quando criança 2  Não 3  Não sabe 4 |
| 39. V. já recebeu alguma vez na vida a vacina anti-tetânica? Quantas doses?  ***(Anote 0 se nunca recebeu; se não lembra quantas recebeu, anote 1)*** TEVIDA | No. de doses ___  Não sabe 9 |
| 40. Vc. já tomou Sulfato Ferroso?  SUF | Sim, recebeu no posto de saúde 1  Sim, comprou na farmácia 2  Não 3  Não sabe 4 |
| 41. Vc. já tomou Ácido Fólico?  ACF | Sim, recebeu no posto de saúde 1  Sim, comprou na farmácia 2  Não 3  Não sabe 4 |
| 42. Alguma vez um médico já lhe disse que V. tinha problema no Útero?  Se Sim, qual problema?  UTER  OUT42 OU42Q | Sim, endometriose 1  Sim, mioma (tumor benigno do útero) 2  Sim, outro tumor do útero 3  Não 4  Outro: ______________________________ 5 |
| 43. V. já fez alguma destas cirurgias?  HI43 Histerectomia (retirada do útero)  PE43 Períneo (reparar a vagina)  MA43 Mastectomia (retirada da(s) mama(s))  TI43 Tireoidectomia (retirada da tireoide) | 1 - Sim 2 – Aguarda fazer 3 – Não  1 - Sim 2 – Aguarda fazer 3 – Não  1 - Sim 2 – Aguarda fazer 3 – Não  1 - Sim 2 – Aguarda fazer 3 – Não |
| 44. Fez exame de prevenção de câncer (ginecológico) nos últimos 12 meses?  PRECA12 | Sim 1  Não 2 |
| 45. Se não, já fez alguma vez na vida?  PRECAVI | Sim 1  Não 2 |
| 46. Seus seios foram examinados em alguma consulta nos últimos 12 meses?  *( Não inclui o exame feito numa consulta de pré-natal)* EXSEIOS | Sim 1  Não 2 |
| 47. V. já fez exames de mamografia? Quantos exames já fez?  ***( Se fez, mas não lembra quantos, anote 1)***  EXMAQ | Sim, ___ exames  Nunca fez 8  Não sabe o que é mamografia 9 |
| 48. Alguma vez na vida V. fez o teste de HIV?  ***(Se Não ou Não Sabe, passe para Q 51)*** EXAIDS | Sim 1  Não 2  Não sabe 3 |
| 49. Se Sim, por que fez este exame?  PQEXAID  *(Ler as opções; marcar mais de uma opção se apropriado)* | Queria saber 1  O médico pediu 2  Estava grávida 3  Doou sangue 4 |
| 50. V. recebeu o resultado deste exame? Na mesma hora (Teste Rápido) ou dias depois? RESEXA  ***(OBS: Não perguntar sobre qual foi o resultado do exame)*** | Sim, na mesma hora 1  Sim, recebeu depois 2  Quanto tempo depois: _________________________  Não recebeu 3 |
| 51. Quantos anos V. tinha quando veio a primeira regra (menstruação)?  ***(Anote 00 se ainda não teve; e passe para Questão 54 )*** PRIMEN | Anos __ __ |
| 52. Quais destes problemas V. costuma ter/sentir quando vem sua menstruação?  ES52 Excesso de sangramento  CD52 Se curvar de tanta dor  CC52 Não cuidar da casa/Não fazer comida  FE52 Faltar ao trabalho/ à escola  SE52 Ter que procurar um serviço de emergência  AN52 Ter que usar analgésico forte/narcótico  AC52 Ter que tomar anticoncepcional para controlar  CR52 Ter que tomar calmante (remédio pros nervos)  OUT52 OU52Q Outro: _____________________________ | 1 - Sim, sempre 2 - Sim, às vezes 3 - Não  1 - Sim, sempre 2 - Sim, às vezes 3 - Não  1 - Sim, sempre 2 - Sim, às vezes 3 - Não  1 - Sim, sempre 2 - Sim, às vezes 3 - Não  1 - Sim, sempre 2 - Sim, às vezes 3 – Não  1 - Sim, sempre 2 - Sim, às vezes 3 – Não  1 - Sim, sempre 2 - Sim, às vezes 3 – Não  1 - Sim, sempre 2 - Sim, às vezes 3 – Não  1 - Sim, sempre 2 - Sim, às vezes 3 – Não |
| 53. Vc. costuma fazer alguma higiene íntima quando vem a menstruação?  Se Sim, qual? HIGI  OUT53 OU53Q | Sim, asseio com água/sabonete 1  Sim, usa absorvente 2  Sim, asseio e absorvente 3  Sim, outro: __________________________ 4  Não faz nada especial 5 |
| 54. V. já teve sua primeira relação sexual? Quantos anos V. tinha na ocasião?  ***(Se ainda não teve, anote 00; 99 se não lembra)*** PRIREL | Anos __ __ |
| **SE AINDA NÃO TEVE RELAÇÃO SEXUAL, PASSE PARA A QUESTÃO 107** | |
| 55. Atualmente V., ou seu companheiro, usam algum método para evitar filhos?  Qual método usa com mais frequência?  METOQ | Coito interrompido 01  Amamentação 02  Tabela 03  Temp.Basal / Billings 04  Diafragma 05  DIU 06  Camisinha masculina 07  Camisinha feminina 08  Pílula 09  Injeção 10  Geléia 11  Vasectomia 12  Outro:________________ 13  Nenhum 14 |
| 56. Se não usa nenhum método, por que não usa?  METPQ | Não é sexualmente ativa 01  Esterilidade 02  Menopausa 03  **Ligação de trompas 04**  Vasectomia 05  Não pode comprar método 06  Não tem método na unidade 07  Não pode usar método 08  Não quer usar método 09  Companheiro não quer 10  Quer engravidar 11  Está grávida 12  Outro: _________________ 13 |
| 57. V. teve alguma relação sexual nos últimos 30 dias?  RELSEX | Sim 1  Não 2  Não quis responder 3 |
| 58. Se Sim, na última relação sexual foi usada a camisinha?  CAMUS | Sim, camisinha masculina 1  Sim, camisinha feminina 2  Não usou 3 |
| 59. Se Não, por que não usou a camisinha?  CAMNUS | Não gosta de usar 1  Não quis usar 2  Parceiro não quis usar 3  Confia no parceiro 4  Não pode comprar 5  Já usa outro método 6  Outro:___________________ 7 |
| 60. V. já ouviu falar, ou já usou, a anticoncepção de emergência (‘pílula do dia seguinte’)? ANTIEM  *(utilizada logo após a relação, quando há risco de engravidar)* | Sim, já ouviu falar 1  Sim, já usou 2  Não 3 |
| 61. V. já ficou grávida alguma vez? GRAVIDA  *(considere gestação que terminou em aborto)* | Sim 1  Não 2 |
| **SE NUNCA ENGRAVIDOU, VÁ PARA A QUESTÃO 107 (Saúde Mental (Depressão))** | |
| 62. Quantas vezes V. já ficou grávida?  *(incluindo gestações que terminaram em aborto)*  GRAVEZ | Gravidezes __ __ |
| 63. Com que idade V. ficou grávida pela primeira vez?  GRAPRIM | Idade __ __ |
| 64. Com que idade V. teve seu primeiro filho?  FILPRIM | Primeiro filho __ __ |
| 65. Quantos filhos V. já teve? (não inclui abortos)  FILTI | Filhos tidos __ __ |
| 66. Destes, quantos nasceram mortos?  *(a partir de 7 meses (ou 28 semanas) de gestação)* NATIM | Filhos mortos __ __ |
| 67. E quantos nasceram vivos?  NATIV | Filhos vivos __ __ |
| 68. Dos filhos que nasceram vivos, quantos estão vivos até hoje?  VIVOS | Vivos hoje __ __ |
| 69. E quantos morreram?  MORTOS | Morreram ___ |

| 70. Dos filhos que nasceram vivos, quantos nasceram de **MAIO de 2014** para cá?  NASC14 | | | Filhos ____ |
| --- | --- | --- | --- |
| 71. Dos filhos que **morreram**: | | | |
| 71a. Qual a data do nascimento? | 71b. Qual a idade quando morreu? | 71c. De que morreu (Causa do óbito)? | |
| U71 Ultimo ___ /___/______    P71 Penul. ___/___/______  A71 Anpen. ___/___/______ | U71M ____ dias ____ meses  P71M ____ dias ____ meses    A71M ____ dias ____ meses | U71C ______________________________________________________  P71C _______________________________________________________  A71C ________________________________________________________ | |

| 72. Teve algum aborto nos últimos 12 meses?  ABORT | Sim 1  ***(Se Não, pular para Q76) 🡪*** Não 2 | |
| --- | --- | --- |
| 73. Se sim, Quantos abortos foram espontâneos? ABESP  E quantos foram provocados? ABPRO | Espontâneos ___  Provocados ___ | |
| 74. Dos abortos espontâneos, em quantos V. precisou ir a um hospital? HESP  Dos abortos provocados, em quantos V. precisou ir a um hospital? HPRO | Espontâneos ___  Provocados ___ | |
| 75. Quais destes problemas Vc. teve no último aborto?  AN75 Anemia  IN75 Infecção  OUT75 OU75Q Outro: ____________________________ | Sim 1 Não 2  Sim 1 Não 2  Sim 1 Não 2 | |
| **VERIFIQUE SE TEVE PARTO NOS ÚLTIMOS 3 ANOS (DE MAIO DE 2014, PRA CÁ), SE NÃO VÁ PARA QUESTÃO 103** | | |
| **76. Qual a data do seu último parto? *(Deve ser igual à Data do Nascimento no Questionário da Criança)*** | | **__ __ / __ __ / __ __ __ __** |
| 77. Na gravidez desse último filho fez quantas consultas pré-natal?  *(Nenhuma consulta=00)* [PNCON] | Nª de consultas __ __ | |
| 78. Com quantos meses de gravidez fez a primeira consulta pré-natal?  [PNPRI] | Meses ___ | |
| 79. Tomou Sulfato Ferroso (para anemia) nesta última gravidez?  [PNFER] | Sim 1  Não 2 | |
| 80. Tomou Ácido Fólico nesta última gravidez?  [PNAFO] | Sim 1  Não 2 | |
| 81. Durante o pré-natal, nesta última gravidez, suas mamas foram examinadas?  [PNMAM] | Sim 1  Não 2 | |
| 82. Durante o pré-natal, nesta última gravidez, foi feito exame ginecológico (interno)? [PNGIN] | Sim 1  Não 2 | |
| 83. Tem Caderneta ou Cartão da Gestante? Pode mostrar?  [CARTAO] | Sim, visto 1  Sim, não visto 2  Não tem 3 | |
| 84. Se mostrar a Caderneta, anotar o No. do Cartão do SUS:  ***(Em”Identificação”, início da Caderneta nova)*** CARSUS | __ __ __ __ __ __ __ __ __ __ __ __ __ __ __ | |
| 85. Qual foi a Data Provável do Parto?  ***(Se Não Tem Caderneta, anotar data informada pela mãe)*** DPP | DPP __ __ / __ __ / __ __ __ __ | |
| 86. Qual foi a Data do Parto?  ***(Se Não Tem Caderneta, anotar data informada pela mãe)*** DPA | DP __ __ / __ __ / __ __ __ __ | |
| 87. Quantas consultas foram feitas por trimestres da gravidez?  [CARTRI]  ***(Se Não Tem Caderneta, anotar informações da mãe)*** | 1o. trim (1-12 sem.) ___  2o. trim (13-24 sem.) ___  3o. trim (25 ou + sem.) ___ | |
| 88. Há pesos anotados no cartão?  [CARPES]  *(observe e anote)* | Sim, em todas as consultas 1  Sim, em algumas consultas 2  Não 3 | |
| 89. Há medidas da Pressão Arterial anotadas no cartão?  [CARPA]  *(observe e anote)* | Sim, em todas as consulta 1  Sim, em algumas consultas 2  Não 3 | |
| 90. Fez quais tipos de exame durante o pré-natal deste último filho?  [PNSAN] Exame de sangue  ***(Leia as opções)***  [PNURI] Exame de urina  *(****Consultar Cartão, se tiver)***  [PNVDRL] Exame VDRL (Sífilis)  [PNHIV] Teste de HIV (Aids)  [PNULS] Exame de ultrassom | 1 - Sim 2 - Não 3 - Não sabe  1 - Sim 2 - Não 3 - Não sabe  1 - Sim 2 - Não 3 - Não sabe  1 - Sim 2 - Não 3 - Não sabe  1 - Sim 2 - Não 3 - Não sabe | |
| 91. Recebeu a vacina anti-tetânica nesta última gravidez? Quantas doses?  PNTET  ***(Anote 0 se não recebeu a vacina)***  *(****Consultar Cartão, se tiver)*** | Sim, No. de doses ___  Não, já tinha recebido 7  Não, nunca recebeu 8  Não sabe 9 | |
| 92. No pré-natal V. recebeu alguma orientação, ou encaminhamento,  sobre o hospital onde deveria ter o seu parto? PARORI | Sim, foi orientada 1  Sim, foi encaminhada 2  Não 3 | |
| 93. Onde foi o parto?  PAROND  QMUN | Hospital do município 1  Hosp. de outro município 2  Qual mun.? ____________________ Em casa 3 | |
| 94. Quem atendeu o parto?  PARATE | Médico 1  Enfermeiro 2  Parteira 3  Outro: __________________________ 4 | |
| 95. Como foi o parto?  PARTIPO | Normal 1  Fórceps 2  Cesárea (1ª. vez que fez uma cesárea) 3  Cesárea (já tinha feito outra cesárea) 4 | |
| 96. Se Cesárea, a cirurgia foi de urgência ou com dia marcado?  CESA | **Urgência** (Já tinha entrado em trabalho de parto) 1  **Urgência** (Ainda não tinha entrado em trabalho de parto) 2  Cesárea com dia marcado 3 | |
| 97. Se foi com dia marcado, quem decidiu que o parto seria Cesárea?  DCESA  OU97Q | O médico sugeriu 1  O médico decidiu 2  Você mesma pediu 3  Você e o médico decidiram 4  Outro: _____________________________ 5 | |
| 98. A criança mamou no peito logo depois do parto?  *(ainda na sala de parto)*  MAPEITO | Sim 1  Não 2 | |

| 99. Você teve algum destes problemas de saúde depois do parto?  POSDOR Dor de cabeça  POSMAM Mamas inflamadas  POSFEB Febre  POSEC Secreção com mau-cheiro  FISTU Fístula (passagem bexiga-vagina)  POSIU Infecção urinária  POSPA Pressão alta  POSCON Convulsão/ataque  POSAMA Problema de amamentação  POSAN Sangramento  POSOUT POSOUQ Outro: ___________________________________ | 1-Sim 2-Não  1-Sim 2-Não  1-Sim 2-Não  1-Sim 2-Não  1-Sim 2-Não  1-Sim 2-Não  1-Sim 2-Não  1-Sim 2-Não  1-Sim 2-Não  1-Sim 2-Não  1-Sim 2-Não |
| --- | --- |
| 100. Fez consulta de revisão do parto durante o resguardo?  ***(Dentro de 45 dias após o parto)*** [POSCON] | Sim 1  Não 2 |
| 101. Foi visitada em casa por alguém da saúde nos primeiros 30 dias após o parto?  [POSDIAS] | Sim, agente de saúde 1  Sim, médico ou enfermeira do PSF 2  Não 3 |
| 102. Durante a gestação, no parto ou no resguardo, recebeu alguma orientação sobre como dar de mamar à criança? ORIGES Na gestação  ORIPAR No parto  ORIPOS No resguardo | 1- Sim 2- Não  1- Sim 2- Não  1- Sim 2- Não |
| **QUESTÕES 103 A 106 SOMENTE PARA MULHERES COM LIGAÇÃO DE TROMPAS (Q56-04). SE NÃO, PASSE PARA Q. 107** | |
| 103. Quantos anos V. tinha quando fez a ligação?  [LIGANOS] | Anos ___ ___ |
| 104. Quantos filhos vivos V. tinha quando fez a ligação?  [LIGAFIL] | Filhos ___ ___ |
| 105. Como foi feita a ligação?  [LIGAPAR] | Na cesariana 1  No pós-parto (logo após o parto) 2  No intervalo (algum tempo depois do parto) 3 |
| 106. Quem tomou a decisão de fazer a ligação?  [LIGADEC] | A própria mulher 1  O casal 2  O companheiro 3  O médico 4 |
|  | |
| **107. Questões sobre Saúde Mental (Depressão) – SRQ20** | |
| 107.1. Você tem dores de cabeça freqüente?  DC107 | Sim 1  Não 2 |
| 107. 2. Tem falta de apetite?  FA107 | Sim 1  Não 2 |
| 107. 3. Dorme mal?  DM107 | Sim 1  Não 2 |
| 107.4. Assusta-se com facilidade?  AF107 | Sim 1  Não 2 |
| 107.5. Tem tremores nas mãos?  TM107 | Sim 1  Não 2 |
| 107.6. Sente-se nervoso (a), tenso (a) ou preocupado (a)?  NT107 | Sim 1  Não 2 |
| 107.7. Tem má digestão?  MD107 | Sim 1  Não 2 |
| 107.8. Tem dificuldades de pensar com clareza?  DP107 | Sim 1  Não 2 |
| 107.9. Tem se sentido triste ultimamente?  ST107 | Sim 1  Não 2 |
| 107.10. Tem chorado mais do que costume?  CH107 | Sim 1  Não 2 |
| 107.11. Encontra dificuldades para realizar com satisfação Suas atividades diárias?  SA107 | Sim 1  Não 2 |
| 107.12. Tem dificuldades para tomar decisões?  TD107 | Sim 1  Não 2 |
| 107.13. Tem dificuldades no serviço (seu trabalho é penoso, causa sofrimento)?  DS107 | Sim 1  Não 2 |
| 107.14. É incapaz de desempenhar um papel útil em sua vida?  PU107 | Sim 1  Não 2 |
| 107.15. Tem perdido o interesse pelas coisas?  PC107 | Sim 1  Não 2 |
| 107.16. Você se sente uma pessoa inútil, sem préstimo?  PI107 | Sim 1  Não 2 |
| 107.17. Tem tido idéia de acabar com a vida?  AV107 | Sim 1  Não 2 |
| 107.18. Sente-se cansado (a) o tempo todo?  CT107 | Sim 1  Não 2 |
| 107.19. Você se cansa com facilidade?  CF107 | Sim 1  Não 2 |
| 107.20. Têm sensações desagradáveis no estomago?  SD107 | Sim 1  Não 2 |

| **Violência doméstica ou de parceiro íntimo:** | |
| --- | --- |
| 108. Você se considera segura em casa?  SEGUR | Sim 1  Não 2 |
| 109. Alguém bate em você ou lhe chama de nomes (xinga)?  ABATE | Sim 1  Não 2 |
| 110. Vc. já empurrou, bateu, chutou, esmurrou um outro adulto?  VBATE | Sim 1  Não 2 |
| 111. Vc. já foi empurrada, espancada, chutada, esmurrada por outro adulto?  OBATE | Sim 1  Não 2 |

| 112. Nos últimos 12 meses, quais destes problemas de saúde V. teve?  DE112 Dengue  ZI112 Zika  CH112 Chikungunya  AL112 Alergia  SI112 Sinusite  PN112 Pneumonia  OUT112 OU112Q Outro: ___________________________ | 1-Sim 2-Não  1-Sim 2-Não  1-Sim 2-Não  1-Sim 2-Não  1-Sim 2-Não  1-Sim 2-Não  1-Sim 2-Não |
| --- | --- |

| **EXAME ANTROPOMÉTRICO DA MULHER** | |
| --- | --- |
| 113. Circunferência da Cintura (CC):  *(Medida logo abaixo das costelas)* CIRCIN | **CC (cm): ___ ___ ___, ___** |
| 114. Circunferência Abdominal (CA):  *(Medida na altura do umbigo)* CIRCAB | **CA (cm): ___ ___ ___, ___** |
| 115. Circunferência do Quadril (CQ):  *(Medida na altura dos glúteos)*  CIRCQD | **CQ (cm): ___ ___ ___, ___** |
| 116. Peso (P):  PESOM | **P (kg): ___ ___ ___, ___** |
| 117. Estatura (E):  ESTATM | **E (cm): ___ ___ ___, ___** |

Entrevistadora: _____________________________________________________________________ Data:___ / ___ / _____

| UFC / UNICHRISTUS / FUNCAP-CNPq  **VI PESQUISA DE SAÚDE MATERNO-INFANTIL NO CEARÁ - 2017** | INFORMAÇÕES DACRIANÇA MENOR DE 6 ANOS |
| --- | --- |

01. Município: ______________________________________ Nome Criança:____________________________

02. [MUN]: ___ ___ [SETOR]:__ __ __ __ ***(4 Últimos Nos. do Mapa)*** [CASA]:__ __ [MULHER]: __ __ No.[CRI]ANÇA: ___

| 03. Qual o sexo de * (a partir daqui falar o nome da criança)?  [SEXO] | | Masculino 1  Feminino 2 | | |
| --- | --- | --- | --- | --- |
| **04. Qual a data de nascimento de * ?**  ***(Deve ser igual à Data do Último Parto (Q76) no Questionário da Mulher,*** DATN  ***Se for o filho mais novo)*** | | | **Data: __ __ /__ __/ __ __ __ __** | |
| 05. Qual a idade de * ?  [IDADE] | | Anos: ___ meses: ___ ___ | | |
| 06. O que a V. é de *?  [RESPOND] | | Mãe biológica 1  Mãe adotiva 2  Avó 3  Tia 4  Irmã 5  Outro: ___________________ 6 | | |
| 07. A mãe de * mora na casa?  [MORAM] | | Sim, a mãe biológica 1  Sim, a mãe adotiva 2  Não a mãe morreu 3  Não mora 4 | | |
| 08. O pai de * mora na casa?  [MORAP]  ***(Se Mora pai biológico ou adotivo, pular para Q12)*** | | Sim, o pai biológico 1  Sim, o pai adotivo 2  Não, mora o padrasto 3  Não o pai morreu 4  Não mora 5 | | |
| 09. Se não mora em casa, o pai biológico vê/fica com a criança?  PAIVE | | Sim, sempre 1  Sim, às vezes 2  Não 3 | | |
| 10. Se não mora em casa, a Sra. recebe alguma ajuda do pai biológico da criança?  PAIAJ | | Sim, pensão alimentícia 1  Sim, outra ajuda 2  Não 3 | | |
| 11. Se não mora em casa, a Sra. gostaria de ver o pai biológico da criança com mais frequência? PAIFR | | Sim 1  Não 2 | | |
| 12. Quando engravidou do último filho V. queria ficar grávida?  [QUERIA] | | Sim, queria 1  Não planejei, mas fiquei feliz 2  Não, queria noutro momento 3  Não queria mais filho 4  Nunca aceitei este filho 5  NS / NQR 6 | | |
| 13. Quais destas pessoas cuidam da criança em casa?  MC13 Mãe  PC13 Pai  AC13 Avó  TC13 Tia  IC13 Irmã  OUC13 OC13Q Outro: _______________________ | | 1 - Sim 2 - Não  1 - Sim 2 - Não  1 - Sim 2 – Não  1 - Sim 2 - Não  1 - Sim 2 - Não  1 - Sim 2 - Não | | |
| 14. A Sra. (a mãe) tirou licença maternidade? Quantos meses?  LIMAT  LIMES | | Sim 1  Meses: ____  Não 2 | | |
| 15. A Sra. (a mãe) tomou Sulfato Ferroso?  SFM | | Sim, antes da gestação 1  Sim, durante a gestação 2  Sim, antes e durante a gestação 3  Não tomou 4  Não sabe 5 | | |
| 16. A Sra. (a mãe) tomou Ácido Fólico?  AFM | | Sim, antes da gestação 1  Sim, durante a gestação 2  Sim, antes e durante a gestação 3  Não tomou 4  Não sabe 5 | | |
| 17. A Sra. (a mãe) fumou durante a gestação da criança?  FUM | | Sim, fumou pouco 1  Sim, fumou moderado 2  Sim, fumou muito 3  Não fumou 4 | | |
| 18. A Sra. (a mãe) bebeu durante a gestação da criança?  BEM  *(bebidas alcoólicas)* | | Sim, bebeu pouco 1  Sim, bebeu moderado 2  Sim, bebeu muito 3  Não bebeu 4 | | |
| 19. A criança vive em outra casa, além desta?  OUCA | | Sim 1  Não 2 | | |
| 20. SE Sim, de quem é a outra casa?  OUCAQ  OUC20 OC20Q | | Da mãe 1  Do pai 2  Dos avós 3  Da tia/tio 4  Da madrinha/padrinho 5  Outro: ___________________ 6 | | |
| 21. O(A) * tem declaração ou certidão de nascimento?  [DECLNAS] | | Sim, declaração 1  Sim, certidão 2  Não 3 | | |
| 22. O(A) * tem a Caderneta da Criança?  [CARTAO]  *(Pedir para ver o Caderneta)* | | Sim, visto 1  Sim, não visto 2  Não, perdeu 3  Nunca teve 4 | | |
| **Informações 22a. e 22b. a serem copiadas da Caderneta da Criança (Ver a Página 39)** | | | | |
| 22a. Dados do Nascimento:  Peso ao nascer: __ __ __ __ g Comprimento ao nascer: __ __ __ cm Perímetro cefálico: __ __ , __ cm *(anote 00 se não preenchido)*  **Apgar**: 1º min: ____ 5º min: ____ Idade gestacional (IG): __ __ semanas __ __ dias | | | | |
| 22b. Testes de Triagem:  Manobra de Ortolani  Teste do Reflexo Vermelho  Teste do Pezinho  Triagem Auditiva | | 1 - Negativo 2 - Positivo 3 - Não preenchido  1 - Normal 2 - Alterado 3 - Não preenchido  1 - Não 2 - Sim 3 - Não preenchido  1 - Não 2 - Sim 3 - Não preenchido | | |
| 23. Quanto a criança pesou ao nascer?  *(Ignorado = 9999)*  [PESONAS]  ***(Perguntar à mãe, se o Peso não estiver anotado na Cardeneta)*** | | Peso ao Nascer Confirmado na Caderneta: ___ ___ ___ ___ g  Peso ao nascer Informado pela Mãe: ___ ___ ___ ___ g | | |
| 24. A gestação foi só desta criança, ou foi de gêmeos?  GEMELAR | | Gravidez simples 1  Gravidez gemelar 2 | | |
| 25. O(A) * nasceu de tempo, antes do tempo ou depois do tempo?  [NASTEMP] | | Nasceu de tempo 1  Nasceu antes do tempo (Prematuro) 2  Nasceu depois do tempo 3  Não sabe 4 | | |
| 25a. Em que posição (na barriga) o bebê estava na hora de nascer?  POSICAO | | Bebê estava de cabeça 1  Bebê estava sentado (de nádegas) 2  O bebê estava atravessado 3  Não sabe 4 | | |
| 26. O bebê ficou internado após o parto?  RNINT | | Sim, num berçário 1  Sim, numa incubadora 2  Sim, numa UTI 3  Não, teve alta com a mãe 4 | | |
| 27. Se Sim, quantos dias ficou internado?  RNINQ | | ___ ___ dias | | |
| 28. Se Sim, o bebê precisou de:  RE28 Ressuscitação  OX28 Oxigênio  AB28 Antibióticos  AS28 Alimentação por sonda  BL28 Banho de luz  CG28 Ser aquecido junto à mãe (Canguru) | | 1 - Sim 2 - Não 3 - Não sabe  1 - Sim 2 - Não 3 - Não sabe  1 - Sim 2 - Não 3 - Não sabe  1 - Sim 2 - Não 3 - Não sabe  1 - Sim 2 - Não 3 - Não sabe  1 - Sim 2 - Não 3 - Não sabe | | |
| 29. A criança mamou na 1ª. hora após nascer?  MAMPH | | Sim 1  Não 2 | | |
| 30. O(A) * mama no peito?  [MAMA] | | Sim 1  Não 2 | | |
| 31. Se Não, até que idade * mamou no peito? [MAMOU]  *(idade em meses)* | | Meses ___ ___  Nunca mamou 77 | | |
| 32. Com que idade * começou a receber: *(idade em meses)* [OUTALIM]    ***(Mencione as opções)*** | | Água ou chá: ___ ___  Outro leite: ___ ___  Mingau (leite+massa): ___ ___  1^as^ comidas sólidas: ___ ___  Comida de panela: ___ ___ | | |
| 33. Quanta doses destas vacinas a criança já tomou? BCG  HEPB  **(*Confira na Caderneta da Criança, pág. 82 (velha), pág. 84 (nova))*** SABIN  PENTA  *(Some e anote o No. de doses de cada vacina)*  ROTAV  PNEUM  *(Anote 0 (zero) se não tomou nenhuma dose)*  MENIN  FEBRA  HEPA  *(Se a mãe diz que tomou, mas não lembra quantas doses, anote 1)* TRIVIR  TETRA  OUTV | | BCG (cicatriz no braço) ___  Hepatite B ___  Anti-Pólio (VIP/VOP) Sabin ___  Pentavalente ___  Rotavírus ___  Pneumocócica ___  Meningocócica C ___  Febre Amarela ___  Hepatite A ___  Tríplice Viral ___  Tetra Viral ___  Outra Vacina: ____________________ | | |
| 34. Fonte de informações sobre as vacinas: [FONTV] | | 1- Caderneta 2- Mãe 3- Ambos | | |
| 35. O(A) * já tomou alguma dose de Vitamina A?  ***(cápsulas de vitamina A)*** [VITA] | | Sim 1  Não 2 | | |
| 36. Se Sim, quantas doses (cápsulas) ele tomou?  **(*Confira na Caderneta da Criança, pág. 81 (velha), pág. 83 (nova))*** DOVA | | Doses: __ | | |
| 37. Se Sim, quando ele(a) tomou estas doses?  DOVAQ  **(*Confira na Caderneta da Criança, pág. 81 (velha), pág. 83 (nova))*** | 1ª. dose: __ __ /__ __/ __ __ __ __  2ª. dose: __ __ /__ __/ __ __ __ __  3ª. dose: __ __ /__ __/ __ __ __ __ 4ª. dose: __ __ /__ __/ __ __ __ __  5ª. dose: __ __ /__ __/ __ __ __ __ | | | 6ª. dose: __ __ /__ __/ __ __ __ __  7ª. dose: __ __ /__ __/ __ __ __ __  8ª. dose: __ __ /__ __/ __ __ __ __ 9ª. dose: __ __ /__ __/ __ __ __ __ |
| 38. O(A) * foi pesada nos últimos 3 meses?  [PESOCAR] | | Sim, registrado cartão 1  Sim, não registrado 2  Não foi pesado 3 | | |
| 39. A * tem algum problema de nascença (congênito)? Qual? PCONG  PCONQ | | Sim 1  Qual? ______________________________________  Não 2 | | |
| 40. A criança já fez alguma cirurgia? Qual? CIRUG  CIRUQ | | Sim 1  Qual? ______________________________________  Não 2 | | |
| 41. A Criança usou antibiótico nos últimos seis meses? Qual? ANTBI  ANTBQ | | Sim 1  Qual? ______________________________________  Não 2 | | |
| 42. O(A) * teve diarréia nas últimas 24 horas?  [DIAR24] | | Sim 1  Não 2 | | |
| 43. O(A) * teve diarréia nos últimos15 dias?  [DIAR15] | | Sim 1  Não 2 | | |
| 44. Você deu algum soro a * para tratar a diarréia?  *(Soro oral rehidratante)* [SORO] | | Sim 1  Não 2 | | |
| 45. A * teve tosse nos últimos 15 dias? [TOS15] | | Sim 1  Não 2 | | |
| 46. Se teve tosse, * tinha dificuldade de respirar? [DIFRES] | | Sim 1  Não 2 | | |
| 47. Por que * tinha dificuldade de respirar?  CAURES  OUC47 OC47Q | | Nariz entupido 1  Cansaço 2  Outro: ___________________3 | | |
| 48. A * tinha febre? [FEBRE]  *(nos últimos 15 dias, quando tinha tosse)* | | Sim 1  Não 2 | | |
| 49. Alguma vez na vida um médico já disse que * tinha Asma? [ASMA] | | Sim 1  Não 2  Não sabe 3 | | |
| 50. A * já teve algum destes acidentes? Qual a gravidade?  QE50 Queda  QM50 Queimadura  EO50 Engolir objetos  TR50 Tomar remédio/veneno  SU50 Sufocação  AF50 Afogamento  CE50 Choque elétrico  AT50 Acidente de trânsito  OUC47 OC47Q Outro: _____________________________ | | 1- Sim, grave 2- Sim, moderado/leve 3- Não  1- Sim, grave 2- Sim, moderado/leve 3- Não  1- Sim, grave 2- Sim, moderado/leve 3- Não  1- Sim, grave 2- Sim, moderado/leve 3- Não  1- Sim, grave 2- Sim, moderado/leve 3- Não  1- Sim, grave 2- Sim, moderado/leve 3- Não  1- Sim, grave 2- Sim, moderado/leve 3- Não  1- Sim, grave 2- Sim, moderado/leve 3- Não  1- Sim, grave 2- Sim, moderado/leve 3- Não | | |
| 51. Se teve algum acidente que considerou grave, como tratou esse acidente?  [ACTRAT] | | Tratou em casa 1  Consultou a criança 2  Hospitalizou a criança 3 | | |
| 52. Nos últimos 3 meses a * fez quantas consultas com um médico? [COMED]  *(Marque 0 se não fez nenhuma consulta)* | | Consultas ___ | | |
| 53. Qual o motivo da última consulta de * com o médico? [COMOT] | | Diarréia 1  Infecções respiratórias 2  Problemas de pele 3  Outra: ___________________ 4  Prevenção: _______________ 5 | | |
| 54. Nos últimos 3 meses a * fez consultas na farmácia, com rezadeiras ou com agentes de saúde? COFARM Farmácia  COREZA Rezadeira COAGEN Agente Saúde | | 1 - Sim 2 - Não  1 - Sim 2 - Não  1 - Sim 2 - Não | | |
| 55. Nos últimos 3 meses a * teve atendimentos com:  FISIO Fisioterapeuta  NUTRIC Nutricionista  TEROC Terapeuta Ocupacional  FONOA Fonoaudióloga PSICOL Psicóloga | | 1 - Sim 2 - Não  1 - Sim 2 - Não  1 - Sim 2 – Não  1 - Sim 2 - Não  1 - Sim 2 - Não | | |
| 56. A * foi internada em hospital nos últimos 12 meses?  *(Marque 0 se não se internou)* [INTERNA] | | Internações ___ | | |
| 57. Se Sim, quantas vezes foi internada por:  [QUINTER]  *(Marque 0 se não se internou por estes motivos)*  OUC57 OC57Q | | Pneumonia ____  Diarréia ____  Dengue ____  Zika ____  Chikungunya ____  Abcesso ____  Outro motivo: __________________________ ____ | | |
| 58. Atualmente a * está indo a uma creche? Gratuita ou paga? [CRECHE] | | Sim, creche pública 1  Sim, creche particular 2  Não 3 | | |
| 59. Atualmente a * está indo a uma Escola? Gratuita ou paga? [ESCO] | | Sim, escola pública 1  Sim, escola particular 2  Não 3 | | |
| 60. Se SIM, quantas horas por dia a * fica na escola ou na creche?  HESC | | Horas ____ | | |
| 61. Quantas horas por dia a * fica assistindo TV?  HTV | | Horas ____ | | |
| 62. Quantas horas por dia a * fica na internet?  HIN | | Horas ____ | | |
| 63. Quantas horas por dia a * fica em aparelhos eletrônicos de toque na tela?  HAE | | Horas ____ | | |
| 64. Quantas horas por dia a * fica em **jogos** eletrônicos (computador/celular/ vídeo-game)? HJE | | Horas ____ | | |
| 65. Quantas horas por dia a * fica brincando sozinha, sem aparelhos eletrônicos?  HBS | | Horas ____ | | |
| 66. Quantas horas por dia a * fica brincando com outras crianças, sem ap. eletrônicos?  HBC | | Horas ____ | | |
| 67. Quantos dias por semana a * tem atividades esportivas?  HAE | | Dias ____ | | |
| 68. Quais destas coisas a * possui?  CE68 Celular  CD68 Computador (desktop ou notebook)  TQ68 TV no próprio quarto  BI68 Bicicleta  FB68 Facebook  OUC68 OC68Q Participa de outras redes sociais: _____________________ | | 1 - Sim 2 - Não  1 - Sim 2 - Não  1 - Sim 2 – Não  1 - Sim 2 - Não  1 - Sim 2 – Não  1 - Sim 2 – Não | | |
| 69. A Sra. já ouviu falar de Autismo, um problema que afeta crianças pequenas?  AUTIS | | Sim 1  Não 2 | | |
| 70. Algum médico já falou para Sra. que a * tem Autismo?  AUTIM | | Sim, faz tratamento 1  Sim, mas não faz tratamento 2  Não 3 | | |
| **As Questões 71.1 a 71.23 sobre Autismo, a seguir, são somente para crianças de 15 a 30 meses de idade.** | | | | |
| 71.1. Seu filho gosta de se balançar, de pular no seu joelho, colo?  BP71 | | Sim 1  Não 2 | | |
| 71.2. Seu filho tem interesse por outras crianças?  IC71 | | Sim 1  Não 2 | | |
| 71.3. Seu filho gosta de subir em coisas, como escadas ou móveis?  SC71 | | Sim 1  Não 2 | | |
| 71.4. Seu filho gosta de brincar de esconder e mostrar o rosto ou de esconde-esconde?  BE71 | | Sim 1  Não 2 | | |
| 71.5. Seu filho já brincou de faz-de-conta, como fazer de conta que está falando no telefone, que está cuidando da boneca, ou outra brincadeira de faz-de-conta? FC71 | | Sim 1  Não 2 | | |
| 71.6. Seu filho já usou o dedo indicador dele para apontar, para pedir alguma coisa?  DP71 | | Sim 1  Não 2 | | |
| 71.7. Seu filho já usou o dedo indicador dele para apontar, para indicar interesse em algo? DI71 | | Sim 1  Não 2 | | |
| 71.8. Seu filho sabe brincar direito com brinquedos pequenos (ex. carros ou blocos), sem apenas colocar na boca, remexer no brinquedo ou deixar o brinquedo cair? BD71 | | Sim 1  Não 2 | | |
| 71.9. O seu filho alguma vez trouxe objetos para você (pais) para lhe mostrar este objeto? TO71 | | Sim 1  Não 2 | | |
| 71.10. O seu filho olha para você no olho por mais de um segundo ou dois?  OO71 | | Sim 1  Não 2 | | |
| 71.11. O seu filho já pareceu muito sensível a barulho (ex. tapando os ouvidos)?  MS71 | | Sim 1  Não 2 | | |
| 71.12. O seu filho sorri em resposta ao seu rosto ou ao seu sorriso?  SR71 | | Sim 1  Não 2 | | |
| 71.13. O seu filho imita você? (ex. você faz expressões/caretas e seu filho imita?)  IV71 | | Sim 1  Não 2 | | |
| 71.14. O seu filho responde quando você chama ele pelo nome?  RN71 | | Sim 1  Não 2 | | |
| 71.15. Se você aponta um brinquedo do outro lado do cômodo, o seu filho olha para ele? OB71 | | Sim 1  Não 2 | | |
| 71.16. Seu filho já sabe andar?  SA71 | | Sim 1  Não 2 | | |
| 71.17. O seu filho olha para coisas que você está olhando?  OC71 | | Sim 1  Não 2 | | |
| 71.18. O seu filho faz movimentos estranhos com os dedos perto do rosto dele?  ME71 | | Sim 1  Não 2 | | |
| 71.19. O seu filho tenta atrair a sua atenção para a atividade dele?  AA71 | | Sim 1  Não 2 | | |
| 71.20. Você alguma vez já se perguntou se seu filho é surdo?  PS71 | | Sim 1  Não 2 | | |
| 71.21. O seu filho entende o que as pessoas dizem?  PD71 | | Sim 1  Não 2 | | |
| 71.22. O seu filho às vezes fica aéreo, “olhando para o nada” ou caminhando sem direção definida? ON71 | | Sim 1  Não 2 | | |
| 71.23. O seu filho olha para o seu rosto para conferir a sua reação quando vê algo estranho? OR71 | | Sim 1  Não 2 | | |

| **Competências Familiares para o Desenvolvimento Infantil** | |
| --- | --- |
| **Estimulação psicossocial:** | |
| 72. Com que frequência as pessoas da casa, inclusive as crianças, almoçam ou jantam todos juntos? ALJU | 1 – Sempre 2 – Às vezes 3 - Nunca |
| 73. Na sua casa tem revistas, livros ou jornais que a criança possa ver?  RELJ | Sim 1  Não 2 |
| 74. A criança tem algum livro infantil ou com figuras ou desenhos?  LINF | Sim 1  Não 2 |
| 75. Todos os dias, vc ou outra pessoa da sua família costuma brincar, ler ou conversar apenas com a criança? FAMB | Sim 1  Não 2 |
| 76. As crianças brincam com muitas coisas diferentes. Eu vou dizer algumas dessas coisas ou brinquedos e gostaria que me dissesse se tem algum em casa para a criança brincar:  BR76 Brinquedos (bola, boneca, bichinho de pelúcia, pião, pipa etc.)  . OC76 Objetos da casa (panelas, colheres, canecos etc.)  OF76 Objetos e materiais de fora da casa (pedras, gravetos etc.)  BT76 Brinquedos que tocam música  BE76 Brinquedos de encaixar, montar  BM76 Brinquedos de movimento, como pular corda, raquetes, chocalho, bambolê  LG76 Lápis, giz, pincel, caneta colorida para escrever/ desenhar | 1 - Sim 2 - Não  1 - Sim 2 - Não  1 - Sim 2 - Não  1 - Sim 2 – Não  1 - Sim 2 - Não  1 - Sim 2 - Não  1 - Sim 2 - Não |
| 77. A criança costuma brincar ou jogar com outras crianças?  BRINC | Sim 1  Não 2 |
| 78. A criança tem contato com animais de estimação da família?  ANIM | Sim, com cachorro 1  Sim, com gato 2  Sim, com gato e cachorro 3  Sim, com outro animal: _______________ 4  Família não cria animal de estimação 5 |
| 79. Na última semana você ou o pai da criança:  PB79 Brincou com ela  PC79 Conversou com ela  PD79 Cuidou dela  PP79 Passeou com ela  PA79 Ajudou/ deu de comer a ela | 1 - Sim 2 – Não  1 - Sim 2 – Não  1 - Sim 2 – Não  1 - Sim 2 – Não  1 - Sim 2 - Não |
| **Apenas para crianças com menos de 1 ano de idade:** | |
| 80. Nos últimos 3 dias, vc. ou outra pessoa da sua família com mais de 15 anos de idade, fez alguma destas coisas com a criança:  BR80 Brincou com brinquedos que fazem sons, barulho, tocam músicas  CA80 Cantou para a criança  JB80 Jogou bola ou objetos que rolam  BP80 Brincou com brinquedos pequenos | 1 - Sim 2 – Não  1 - Sim 2 – Não  1 - Sim 2 – Não  1 - Sim 2 – Não |
| **Apenas para crianças com idade de 1 a 2 anos:** | |
| 81. Nos últimos 3 dias, vc. ou outra pessoa da sua família com mais de 15 anos de idade, fez alguma destas coisas com a criança:  CA81 Cantou  JB81 Jogou bola  BP81 Brincou com brinquedos pequenos  DP81 Desenhou/pintou  PA81 Levou a criança para passear | 1 - Sim 2 – Não  1 - Sim 2 – Não  1 - Sim 2 – Não  1 - Sim 2 – Não  1 - Sim 2 – Não |
| **Apenas para crianças com idade de 3 a 4 anos:** | |
| 82. Nos últimos 3 dias, vc. ou outra pessoa da sua família com mais de 15 anos de  idade, fez alguma destas coisas com a criança: CO82 Correu com a criança  CA82 Cantou para a criança  JB82 Jogou bola  BP82 Brincou com brinquedos pequenos  DP82 Desenhou/pintou  PA82 Levou a criança para passear  QB82 Brincou com quebra-cabeça | 1 - Sim 2 – Não  1 - Sim 2 – Não  1 - Sim 2 – Não  1 - Sim 2 – Não  1 - Sim 2 – Não  1 - Sim 2 – Não  1 - Sim 2 – Não |
| **Apenas para crianças com idade de 4 a 5 anos:** | |
| 83. Você ou outra pessoa da sua família, faz alguma destas coisas com a criança:  EA83 Ensina o alfabeto (as letras)  EL83 Ensina a ler ou escrever  ES83 Ensina a criança sobre como sair com outras crianças  EC83 Ensina a criança sobre como se comportar na escola  AB83 Anda de bicicleta, cavalo etc. | 1 - Sim 2 – Não  1 - Sim 2 – Não  1 - Sim 2 – Não  1 - Sim 2 – Não  1 - Sim 2 – Não |

| **Gerenciamento do comportamento (Questões para todas as crianças):** | |
| --- | --- |
| 84. Quando Vc. sai de casa quem costuma ficar cuidando da criança:  QSAI | Avó/avô da criança 1  Pai da criança 2  Tia/tio da criança 3  Criança < 10 anos 4  Criança > 10 anos 5  Vizinhos 6  Vai junto 7  Fica sozinho 8  Outro: _______________________ 9 |
| 85. O que vc. faz quando a criança não se comporta bem, ou seja, faz alguma coisa que vc. não quer?  RE85 Repreende a criança  CA85 Dá um castigo  FA85 Faz ameaças  BA85 Bate nela  EX85 Diz não e explica porque ela não deve se comportar assim  PA85 Dá umas palmadas  GR85 Grita com a criança  DN85 Diz ‘Não’ ou ‘Pára’  DI85 Distrai a criança  SE85 Manda a criança sentar, ir pro quarto, ou leva ela embora se não está em casa | 1- Sim, espontâneo 2- Sim, induzido 3- Não  1- Sim, espontâneo 2- Sim, induzido 3- Não  1- Sim, espontâneo 2- Sim, induzido 3- Não  1- Sim, espontâneo 2- Sim, induzido 3- Não  1- Sim, espontâneo 2- Sim, induzido 3- Não  1- Sim, espontâneo 2- Sim, induzido 3- Não  1- Sim, espontâneo 2- Sim, induzido 3- Não  1- Sim, espontâneo 2- Sim, induzido 3- Não  1- Sim, espontâneo 2- Sim, induzido 3- Não  1- Sim, espontâneo 2- Sim, induzido 3- Não |
| 86. Quais destas situações acontecem com a criança?  DO86 Criança dorme na mesma cama que os pais  FO86 O lugar onde a criança dorme fica perto do fogão, vela etc.  CH86 O lugar onde a criança dorme fica próximo ao chão  TO86 As tomadas elétricas ficam destampadas  OB86 Os objetos pequenos ficam ao alcance da criança  SO86 A criança fica sozinha  FC86 Deixa a criança ir pra fora de casa  PA86 Os cabos das panelas ficam pra fora do fogão  ME86 Os medicamentos/remédios ficam ao alcance da criança  DE86 Detergente, veneno, água sanitária ficam alto, longe da criança  FA86 Facas e tesouras ficam guardadas/ longe do alcance da criança  BF86 Crianças podem brincar com fogos de artifício e fogueiras | 1 - Sim 2 – Não  1 - Sim 2 – Não  1 - Sim 2 – Não  1 - Sim 2 – Não  1 - Sim 2 – Não  1 - Sim 2 – Não  1 - Sim 2 – Não  1 - Sim 2 – Não  1 - Sim 2 – Não  1 - Sim 2 – Não  1 - Sim 2 – Não  1 - Sim 2 – Não |

| **(QUESTÕES SEGUINTES COM AS PERGUNTAS DO ASQ3)** |
| --- |

| **EXAME ANTROPOMÉTRICO DA CRIANÇA** | |
| --- | --- |
| 87. Peso da Mãe **COM** a Criança: [PESOMC] | P (kg): ___ ___ ___, ___ |
| 88. Peso da Mãe **SEM** a Criança: [PESOM1] | P (kg): ___ ___ ___, ___ |
| 89. Estatura (E): [ESTATC] | E (cm): ___ ___ ___, ___ |
| 90. A criança foi medida: [POSICAO] | 1- Em pé 2- Deitada |
| 91. Perímetro cefálico: [PERICEF] | PC (cm): ___ ___, ___ |

Entrevistadora: _______________________________________________________________________ Data: ___/___/___
